# Pan-lysyl oxidase inhibition disrupts fibroinflammatory tumor stroma, rendering cholangiocarcinoma susceptible to chemotherapy

**DOI:** 10.1097/HC9.0000000000000502

**Published:** 2024-08-05

**Authors:** Paul R. Burchard, Luis I. Ruffolo, Nicholas A. Ullman, Benjamin S. Dale, Yatee A. Dave, Bailey K. Hilty, Jian Ye, Mary Georger, Rachel Jewell, Christine Miller, Luis De Las Casas, Wolfgang Jarolimek, Lara Perryman, Matthew M. Byrne, Anthony Loria, Chelsea Marin, Mariana Chávez Villa, Jen Jen Yeh, Brian A. Belt, David C. Linehan, Roberto Hernandez-Alejandro

**Affiliations:** 1 Department of Surgery, University of Rochester Medical Center, Rochester, New York, USA; 2 Jacobs School of Medicine and Biomedical Sciences, Buffalo, New York, USA; 3 Department of Pathology, The University of Texas Southwestern Medical Center, Dallas, Texas, USA; 4 Drug Discovery, Syntara Ltd., Sydney, New South Wales, Australia; 5 Departments of Surgery and Pharmacology, Lineberger Comprehensive Cancer Center, University of North Carolina System, Chapel Hill, North Carolina, USA; 6 Department of Surgery, Division of Surgical Oncology, University of Rochester Medical Center, Rochester, New York, USA; 7 Division of Solid Organ Transplant Surgery, Department of Surgery, University of Rochester Medical Center, Rochester, New York, USA

## Abstract

**Background:**

Cholangiocarcinoma (CCA) features highly desmoplastic stroma that promotes structural and functional resistance to therapy. Lysyl oxidases (LOX, LOXL1–4) catalyze collagen cross-linking, thereby increasing stromal rigidity and facilitating therapeutic resistance. Here, we evaluate the role of lysyl oxidases in stromal desmoplasia and the effects of pan-lysyl oxidase (pan-LOX) inhibition in CCA.

**Methods:**

Resected CCA and normal liver specimens were analyzed from archival tissues. Spontaneous and orthotopic murine models of intrahepatic CCA (iCCA) were used to assess the impact of the pan-LOX inhibitor PXS-5505 in treatment and correlative studies. The functional role of pan-LOX inhibition was interrogated through in vivo and ex vivo assays.

**Results:**

All 5 lysyl oxidases are upregulated in CCA and reduced lysyl oxidase expression is correlated with an improved prognosis in resected patients with CCA. Spontaneous and orthotopic murine models of intrahepatic cholangiocarcinoma upregulate all 5 lysyl oxidase isoforms. Pan-LOX inhibition reversed mechanical compression of tumor vasculature, resulting in improved chemotherapeutic penetrance and cytotoxic efficacy. The combination of chemotherapy with pan-LOX inhibition increased damage-associated molecular pattern release, which was associated with improved antitumor T-cell responses. Pan-LOX inhibition downregulated macrophage invasive signatures in vitro, rendering tumor-associated macrophages more susceptible to chemotherapy. Mice bearing orthotopic and spontaneously occurring intrahepatic cholangiocarcinoma tumors exhibited delayed tumor growth and improved survival following a combination of pan-LOX inhibition with chemotherapy.

**Conclusions:**

CCA upregulates all 5 lysyl oxidase isoforms, and pan-LOX inhibition reverses tumor-induced mechanical forces associated with chemotherapy resistance to improve chemotherapeutic efficacy and reprogram antitumor immune responses. Thus, combination therapy with pan-LOX inhibition represents an innovative therapeutic strategy in CCA.

## Graphical abstract

**Figure:**
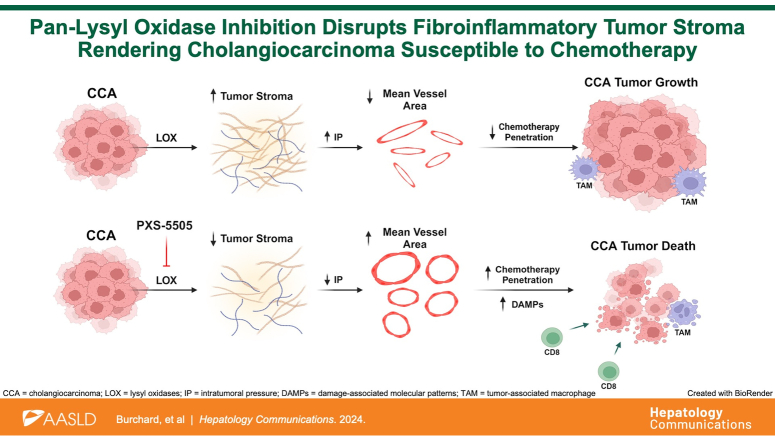

## INTRODUCTION

Cholangiocarcinoma (CCA) is the second most frequently diagnosed primary liver malignancy, and its incidence has nearly doubled over the last 2 decades.^1^ Despite growing prevalence, 5-year survival for both intrahepatic and extrahepatic CCA remains poor at 9%–10%,^2^^,^^3^ with the majority of patients relying on gemcitabine and fluoropyrimidine-based chemotherapy regimens resulting in limited efficacy.^3^^–^^5^ While immunotherapy has recently shown promise,^6^ there remains a significant need for additional systemic treatment strategies that can further improve CCA outcomes.

The tumor microenvironment (TME) of CCA features an expansive desmoplastic reaction composed of dense collagen scaffolds infiltrated with an abundance of fibroblasts and inflammatory immune cells.^7^^,^^8^ While these components provide the underlying framework of the tumor stroma that confers structural and functional resistance to therapy,^7^^,^^9^^,^^10^ the key pathways supporting their development in CCA tumorigenesis remain less clear.

Lysyl oxidases (LOX) are a family of 5 secreted copper-dependent amine oxidases (LOX, LOXL1–4) that catalyze collagen and elastin fibrillogenesis.^11^ All 5 LOX isoforms have been shown to promote oncogenesis in solid tumors through various mechanisms, such as increasing stromal density, facilitating immune evasion, as well as augmenting tumor growth and metastatic spread.^12^^–^^17^ Furthermore, elevated LOX isoform expression correlates with poor prognosis across a variety of solid malignancies, including CCA.^17^^–^^20^ Despite promising preclinical results, efforts to target individual LOX isoforms have failed to achieve clinical impact, likely due to the compensatory action of other LOX family members.^21^^,^^22^ PXS-5505 represents a novel, first-in-class, small-molecule inhibitor of all 5 LOX isoforms and has demonstrated efficacy in mouse models of pancreatic cancer.^23^^,^^24^

Here, we evaluate the role of LOXs in promoting CCA stromal desmoplasia and the clinical potential of pan-LOX inhibition for treating the disease. Utilizing PXS-5505, in combination with chemotherapy, we demonstrate restrained tumor growth and improved survival in orthotopic and autochthonous mouse models of intrahepatic cholangiocarcinoma (iCCA), respectively. Mechanistically, we show that pan-LOX inhibition improves chemotherapeutic penetration and reduces the fibroinflammatory reaction of iCCA, thereby enhancing antitumor immunity.

## METHODS

### Human CCA tumor specimens

Freshly resected snap-frozen human CCA and adjacent normal liver specimens, as well as archival tissue blocks of resected human CCA and nonadjacent normal liver, were obtained through approved Institutional Review Board protocols. Archival “normal” liver tissue blocks were derived from patients undergoing surgery for a benign or malignant mass of the liver and were pathologically scored normal. Demographic and clinicopathologic data were abstracted from the electronic medical record. All research was conducted in accordance with both the Declarations of Helsinki and Istanbul. Written consent was given by all subjects in writing.

### Autochthonous and orthotopic murine models of CCA

The LSL-Kras^G12D^; Tp53^Flox/Flox^; Alb-Cre (KPPC) murine model of iCCA backcrossed to C57BL/6 was previously described.^25^ KPPC mice fed a 3,5-diethoxycarbonyl-1,4-dihydrocollidine-based diet develop primary liver tumors in which the majority exclusively display histopathology consistent with iCCA.^26^ 4-week-old KPPC mice and littermate controls were fed a dietary course of 0.1% 3,5-diethoxycarbonyl-1,4-dihydrocollidine in nutritionally complete pellets (Custom Animal Diets) for 4 weeks, and disease onset was monitored by ultrasound (US) with the Vevo 3100 Imaging System (FUJIFILM VisualSonics). On disease detection, KPPC mice were serially enrolled into treatment cohorts of fluorouracil plus oxaliplatin (FOX) (75 mg/kg 5-fluorouracil plus 2 mg/kg oxaliplatin, i.p. weekly) and nutritionally complete chow (Research Diets) formulated with or without the small-molecule pan-LOX inhibitor PXS-5505 (Syntara Ltd) at 1120 ppm. iCCA histopathology was confirmed postmortem in all available tumors by a board-certified pathologist.

The URCCA4.3 syngeneic murine tumor cell line was previously described^25^ and maintained in low passage numbers in complete medium (CM) containing RPMI 1640 (Gibco), 10% fetal bovine serum (HyClone), and 1X Penicillin-Streptomycin (Gibco). For orthotopic studies, 80,000 URCCA4.3 cells mixed 2:1 in a PBS:Matrigel Matrix Basement Membrane HC (Corning) solution was injected into the left hepatic lobe of 6- to 8-week-old C57BL/6 mice (Jackson Laboratory). After confirmation of disease onset by US 2 weeks following implantation, mice bearing orthotopic URCCA4.3 tumors were randomized into treatment cohorts of vehicle diet, PXS-5505 medicated diet, and FOX with or without the PXS-5505 medicated diet. For T-cell depletion studies, mice were dosed i.p. with 500 µg of anti-CD8 (clone 2.43) and anti-CD4 (clone GK1.5) neutralizing IgG antibodies (BioXcell) prior to tumor implantation followed by 250 µg of each antibody every 4–5 days.

All animal studies were approved by the University Committee on Animal Resources.

### Histology, immunohistochemistry, and immunofluorescence analysis

Mouse tumor and normal liver tissues were fixed in 10% neutral-buffered formalin-fixed paraffin-embedded. Formalin-fixed paraffin-embedded blocks of archived human specimens and mouse tissues were sectioned at 5 μm. Hematoxylin and eosin staining was performed using standard protocols, and Picro-Sirius Red (Polysciences Inc.) and Movats Pentachrome (Poly Scientific R&D Corp.) staining was performed per manufacturer protocol.

For immunohistochemistry (IHC) analysis, endogenous peroxidase activity was quenched with 3% hydrogen peroxide and heat-induced antigen retrieval with citrate (bioWORLD) or high pH (Invitrogen) buffers was performed. Serum-Free Protein Block (Agilent) was used to block nonspecific background and slides were incubated with primary antibodies (Supplemental Table S1, http://links.lww.com/HC9/A992) diluted in Antibody Diluent (Agilent) overnight at 4 °C. Antibody staining was visualized using Polink-2 Plus HRP Broad Bulk and 3,3′-Diaminobenzidine kits (GBI Labs). For LOX isoform staining, slides were incubated with biotinylated horse anti-rabbit IgG (Vector Laboratories) secondary antibody at room temperature, and staining was visualized with Vectastain ABC-HRP (Vector Laboratories) and Liquid 3,3′-Diaminobenzidine+ (Agilent) kits.

For immunofluorescence analysis, heat-induced antigen retrieval was performed with citrate buffer, and slides were blocked and incubated in primary antibodies (Supplemental Table S1, http://links.lww.com/HC9/A992) as described above. Slides were washed with PBST (1X PBS + 0.1% Tween 20) and stained with Alexa Fluor 488 goat anti-rabbit and Alexa Fluor 555 goat anti-mouse IgG secondary antibodies (ThermoFisher) diluted in Antibody Diluent at room temperature. Slides were washed in PBST and coverslips were mounted with Vectashield Vibrance Antifade Mounting Medium with DAPI (Vector Laboratories).

Brightfield and fluorescent images were captured with a BX43 microscope equipped with a DP80 camera (Olympus). For quantitative analysis of IHC staining, tissue sections were scanned with an Aperio VERSA slide scanner and staining was quantified using Aperio Positive Pixel Count and Microvessel algorithms (Leica Microsystems).

### Intratumoral pressure assessment

Prior to sacrifice, mice underwent laparotomy to expose URCCA4.3 tumors. Freshly resected human iCCA tumors and normal liver specimens were obtained postsurgery under an Institutional Review Board–approved protocol. Tissue was punctured with a 27G needle to introduce a Millar SPR-671 Mikro-Tip catheter pressure transducer connected to a FE221 Bridge Amp and PowerLab 4/35 recording unit, and pressure measurements were recorded with LabChart software (ADInstruments).

### Determination of intratumoral chemotherapy concentration

URCCA4.3 tumor-bearing mice were treated with FOX (i.v. weekly) and fed chow with or without PXS-5505 for 4 weeks. Mice were sacrificed 2 hours following the last dose of FOX, and resected tumors were snap frozen. Intratumoral 5-fluorouracil (5FU) concentration was determined using a ultra-performance liquid chromatography-mass spectrometry method performed by Creative Proteomics (Shirley, NY).

### Flow cytometry analysis

Resected URCCA4.3 tumor specimens were minced and processed with a gentleMACs Dissociator into single-cell suspensions in C Tubes (Miltenyi Biotec) containing an enzymatic digest solution for 30 minutes at 37 °C. Cells suspended in flow cytometry staining buffer were blocked with TruStain FcX Antibody (BioLegend Inc.) and stained with panels of fluorophore-conjugated antibodies (Supplemental Table S2, http://links.lww.com/HC9/A992) using standard protocols. For FOXP3 intranuclear staining, the True-Nuclear Transcription Factor Buffer Set (BioLegend Inc.) was used per manufacturer protocol. Sample acquisition was performed on an LSRII (BD Biosciences), and data sets were analyzed with FlowJo software (v.10.6.1).

### Bulk tissue RNA extraction and qRT-PCR analysis

Tumor and adjacent normal liver tissue were homogenized in Trizol (ThermoFisher) using a TissueLyser LT (Qiagen), and total RNA was isolated with RNeasy Mini Kits (Qiagen). Bulk RNA was reverse transcribed into cDNA using High-Capacity RNA-to-cDNA Kits, and quantitative real-time PCR (qRT-PCR) analysis was performed with predesigned TaqMan Gene Expression Assays (Supplemental Table S3, http://links.lww.com/HC9/A992) and TaqMan Fast Universal PCR Master Mix (ThermoFisher) per manufacturer protocol using a CFX96 Touch Real-Time PCR Detection System (Bio-Rad). Gene expression levels were determined with CFX Maestro Software v2.0 (Bio-Rad) using the comparative CT (ΔΔCT) method, and target gene expression was normalized to GAPDH or HPRT1.

For macrophage invasion assay qRT-PCR, cDNA synthesis was performed as described above, and select genes were preamplified with TaqMan PreAmp Master Mix (ThermoFisher) and predesigned TaqMan Gene Expression Assays for 14 cycles following manufacturer protocol. qRT-PCR analysis was performed with diluted preamplification products as described above.

### RNA-sequencing and pathway analysis

RNA-sequencing and bioinformatics analyses were performed by Medgenome. Specifically, sequencing libraries were generated from RNA isolated from upper and lower chamber macrophages of invasion assays described above using the Takara SMARTer Stranded Total RNA-Seq Kit (v2 - Pico Input Mammalian), and sequencing was performed with the NovaSeq platform (Illumina). RNA-seq reads were aligned to the reference mouse genome GRCm38 using STAR (v2.73a), and raw read counts were estimated with HTSeq (v0.11.2). Differential gene expression analysis was performed using DESeq2 (R Bioconductor Package) to determine significant changes (*p*<0.05 and fold change≥1 or≤−1) in differentially expressed protein-coding genes (DEGs). Over-representation analysis to determine the enrichment of Gene Ontology (GO) terms with *p*<0.05 was performed using all DEGs with ClusterProfiler (v3.10.1).

### URCCA4.3 cell line and macrophage chemoresistance assay

Bone-marrow-derived macrophages (BM-DM) were cultured in a 1:1 mixture of CM:tumor-conditioned media (TCM) for 24 hours to generate tumor-associated macrophages (TAMs). 10,000 URCCA4.3 cells or 100,000 TAM were seeded in Nunc F96 MicroWell White Polystyrene Plates (ThermoFisher) layered with Cultrex Basement Membrane Extract (R&D Systems) and cultured in CM or CM:TCM (1:1), respectively, with or without 1 mM PXS-5505 for 24 hours prior to adding fluorouracil and incubating an additional 24 hours. URCCA4.3 and TAM viability were assessed using CellTiter-Glo 2.0 Cell Viability Assays (Promega), and bioluminescence was measured with a Biotek Synergy HTX Multimode Reader.

### Statistical analysis

Continuous and categorical variables were tested for differences using the Mann-Whitney *U* and χ^2^ tests, respectively. Paired patient iCCA tumor specimens and adjacent normal liver were compared through the Wilcoxon matched-pairs signed rank test. Tumor growth was modeled and compared through simple linear regression. Kaplan-Meier survival curves were compared through the log-rank (Mantel-Cox) test with maximal significance cutoffs determined by the survminer package (The Comprehensive R Archive Network). Patients with death within 30 days of surgery were excluded. Statistical analysis was performed on GraphPad Prism (version 9.4.1) or SAS software (version 9.4). Statistical significance was set at *p*<0.05.

## RESULTS

### CCA stromal components correlate inversely with survival

**FIGURE 1:**
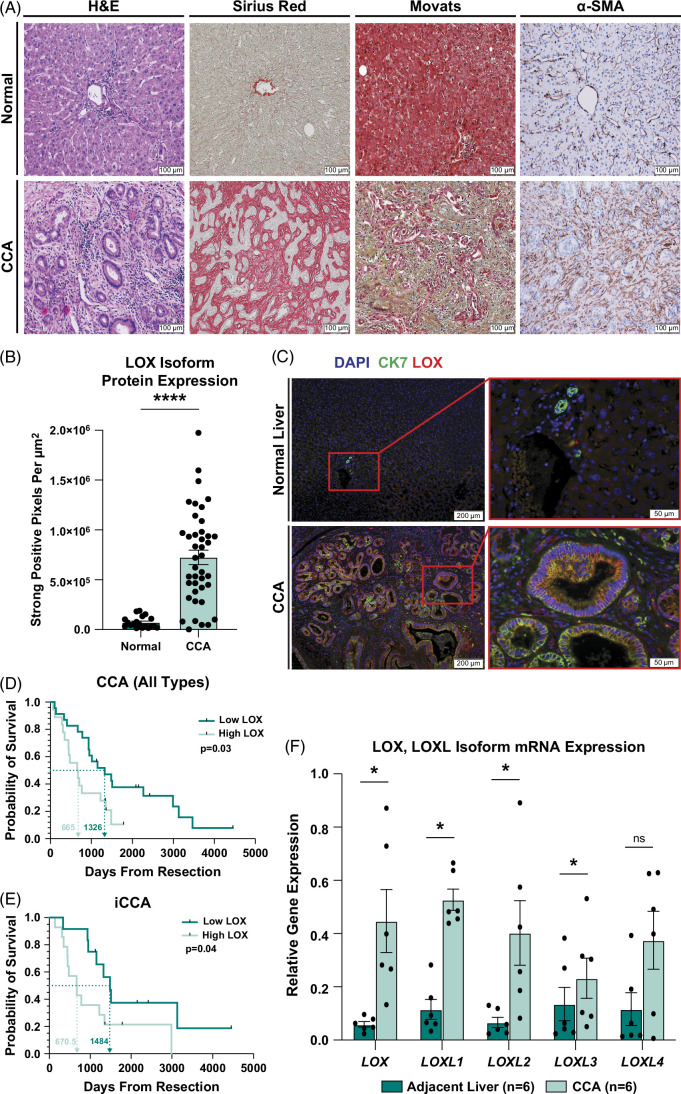
Lysyl oxidases are highly expressed by human CCA, and LOX isoform expression correlates with patient outcomes. (A) Representative images compare H&E, sirius red (red = collagen), Movat pentachrome (yellow= collagen, blue = glycosaminoglycans/mucins, green = collagen and glycosaminoglycan/mucin colocalization, red = malignant biliary epithelium or normal liver parenchyma, and black = nuclei), and α-SMA immunohistochemistry staining in human iCCA versus normal liver tissue sections. Images acquired at ×200 magnification. (B) Graph compares the quantification of LOX isoform expression in archival normal human liver tissue versus resected CCA tumors. Graph depicts mean ±SEM with data points representing whole-section quantification of LOX isoform staining per tissue specimen. *p*-value determined by Mann-Whitney *U* test. **** = *p*<0.0001. (C) Representative images show immunofluorescence staining for nuclei (DAPI = blue), cytokeratin 7 (CK7 = green), and LOX isoforms (LOX = red) in normal human liver and resected CCA tumor specimens. Images were acquired at ×100 and ×400 magnification. (D) Kaplan- Meier curve compares overall survival after resected CCA tumor specimens were stratified into low (n=23) versus high (n=18) LOX isoform staining cohorts. Dashed lines indicate median overall survival in low (blue) versus high (red) staining cohorts. The *p*-value was determined by the log-rank (Mantel-Cox) test with maximal significance cutoffs determined by the survminer package (The Comprehensive R Archive Network). (E) Resected iCCA tumor specimens stratified into low (n=14) versus high (n=12) LOX isoform staining cohorts were compared for overall survival by Kaplan-Meier analysis. Dashed lines indicate median overall survival in low (blue) versus high (red) staining cohorts. The *p*-value was determined by the log-rank (Mantel-Cox) test. (F) Graph compares mRNA expression levels for LOX and lysyl oxidase-like 1-4 isoforms in adjacent normal livers (n=6) versus CCA tumors (n=6) in freshly resected patient specimens. Bar graphs depict mean ±SEM and *p*-values determined by the Wilcoxon matched-pairs signed rank test. *p<0.05. Abbreviations: CCA, cholangiocarcinoma; H&E, hematoxylin and eosin; iCCA, intrahepatic cholangiocarcinoma; LOX, lysyl oxidases; LOXL, lysyl oxidase-like; α-SMA, alpha-smooth muscle actin.

CCA is characterized by highly desmoplastic stroma composed of abundant collagens, glycosaminoglycans, and activated fibroblasts, encompassing malignant biliary epithelium (Figure 1A). In line with previous CCA studies,^27^ whole-section quantification for collagen and activated fibroblast markers within our patient cohort with CCA revealed that resected CCA tumors expressed significantly higher levels of sirius red and alpha-smooth muscle actin (α-SMA) staining compared to normal liver parenchyma, irrespective of CCA subtype (Supplemental Figure S1A–C, http://links.lww.com/HC9/A993, Supplemental Table S4, http://links.lww.com/HC9/A992). Notably, both the extent of collagen deposition and density of activated fibroblasts correlated inversely with overall survival following CCA resection (Supplemental Figure S1D, E, http://links.lww.com/HC9/A993), suggesting these stromal components have important prognostic implications. Similar to the TME of pancreatic cancer, where desmoplastic features have been shown to induce higher intratumoral pressure (IP) associated with therapeutic resistance,^28^^,^^29^ we too observed significant elevation of IP in freshly resected human CCA tumors compared to uninvolved normal liver (Supplemental Figure S2A, http://links.lww.com/HC9/A993).

### All LOX isoforms (LOX, LOXL1–4) are upregulated across CCA subtypes, and LOX isoform expression is prognostic in CCA

LOXs are known to promote desmoplasia in solid tumors and correlate positively with multiple stromal markers, including COL1A1, COL1A2, and α-SMA in CCA (Supplemental Figure S3A–O, http://links.lww.com/HC9/A993).^11^ Therefore, we sought to investigate the potential role of LOX isoforms in CCA tumorigenesis and their clinical relevance. To assess the clinical relevance of LOXs across human CCA, we performed IHC analysis for LOX isoform expression in whole sections of resected CCA tumors and normal liver from archival tissue at our institution. Quantification of IHC staining showed that CCA tumors expressed significantly higher levels of LOX isoform compared to normal liver, irrespective of tumor subtype (Figure 1B, supplemental methods, Supplemental Figure S2B, http://links.lww.com/HC9/A993) with 51.5% of LOX expression localized to the biliary epithelium compared to 36.4% in the surrounding stroma (Figure 1C, Supplemental Figure S2C, http://links.lww.com/HC9/A993). Furthermore, LOX isoform expression levels in resected CCA patient tumors were prognostic and correlated inversely with overall survival (Figure 1D). The majority of these tumor specimens were derived from iCCA, and LOX isoform expression maintained its prognostic value in this cohort (Figure 1E). While multivariate analysis of our data set was unable to confirm LOX isoform significance alone (Supplemental Tables S5, S6, http://links.lww.com/HC9/A992), cox regression of multiple data sets confirmed its relevance in both CCA and iCCA (Supplemental Figure S4, http://links.lww.com/HC9/A993). Notably, qRT-PCR analysis of freshly resected human specimens revealed upregulation of all 5 LOX isoforms (*LOX* and *LOXL1–4*, collectively referred to as pan-LOX hereafter) within human CCA compared to adjacent normal liver, suggesting each plays a key role in shaping the CCA TME and emphasizing the importance of simultaneously targeting all isoforms (Figure 1F). Furthermore, the degree of LOX expression corresponded positively with leukocyte and CD15+ myeloid cell infiltrate, providing a potential link to the tumor immune microenvironment of CCA (Supplemental Figure S5A, B, http://links.lww.com/HC9/A993).

### The KPPC murine model of iCCA recapitulates the desmoplastic features of human disease and upregulates LOX expression

**FIGURE 2:**
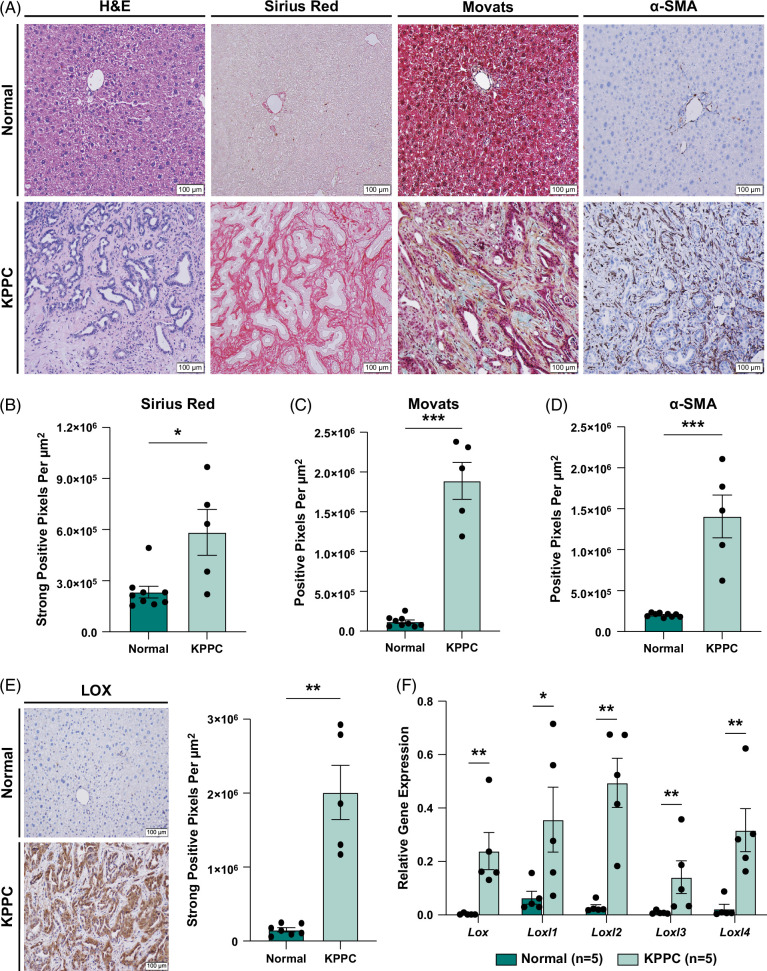
KPPC intrahepatic cholangiocarcinoma tumors feature prominent desmoplasia mirroring human disease and express significantly elevated levels of lysyl oxidases. (A) Representative images show H&E, sirius red, Movat pentachrome, and α-SMA staining in tissue sections from normal livers of littermate controls and KPPC intrahepatic cholangiocarcinoma tumors at the end of life (EOL). Four-week-old KPPC mice and littermate controls were each fed a dietary course of 0.1% DDC in nutritionally complete pellets (Custom Animal Diets) for 4 weeks. EOL was defined as mice that appeared clinically moribund (body condition score <2) and/or those with gross ascites. Images were acquired at ×200 magnification. Intrahepatic cholangiocarcinoma histopathology was confirmed postmortem in all available KPPC tumors by a board-certified pathologist. (B–D) Graphs compare the quantification of sirius red (B), Movat pentachrome (C), and α-SMA immunohistochemistry (IHC) (D) staining in normal livers of littermate controls (n=9) versus KPPC tumors (n=5) at EOL. Data points represent whole-section quantification of IHC staining per tissue specimen. (E) Representative images compare IHC staining for LOX isoforms in normal livers of littermate controls versus KPPC tumors at EOL. The graph shows the quantification of LOX isoform IHC staining in normal livers of littermate controls (n=9) versus KPPC tumors (n=5) at EOL. Data points represent whole-section quantification of LOX isoform staining per tissue specimen. (F) Graph compares mRNA expression levels for Lox and Loxl 1–4 isoforms in normal livers of littermate controls (n=5) versus KPPC tumors (n=5) at EOL by qRT-PCR. Bar graphs depict mean ±SEM and *p*-values determined by Mann-Whitney *U* test. * = *p*<0.05, ** = *p*<0.01, and *** = *p*<0.001. Abbreviations: DDC, 3,5-diethoxycarbonyl-1,4-dihydrocollidine; H&E, hematoxylin and eosin; KPPC, LSL-Kras^G12D^; Tp53^Flox/Flox^; Alb-Cre; LOX, lysyl oxidases; LOXL, lysyl oxidase-like; α-SMA, alpha-smooth muscle actin.

We previously demonstrated that KPPC iCCA tumors have a prominent immunosuppressive milieu, mirroring human disease.^25^ To validate the KPPC mouse model of iCCA for studying other stromal components, we examined the prominence of extracellular matrix and fibroblast markers in murine iCCA tumors. Quantification of sirius red, Movat, and α-SMA staining demonstrated expansive deposition of collagen, glycosaminoglycans, and activated fibroblasts, respectively, in KPPC tumors compared to normal livers of littermate controls (Figure 2A–D, Supplemental Figure S6, http://links.lww.com/HC9/A993). Taken together, these data show the KPPC mouse model faithfully reproduces other prominent features of human disease supporting its utility for studying iCCA stromal biology and evaluating novel strategies for targeting these components.

In line with our human CCA specimens, whole-section analysis of IHC staining demonstrated significantly elevated levels of LOX isoform expression in KPPC tumors compared to normal livers of littermate controls (Figure 2E, Supplemental Figure S6, http://links.lww.com/HC9/A993) with expression patterns that reflected human disease. Furthermore, like human iCCA, qRT-PCR analysis showed that mRNA for all 5 LOX isoforms were significantly upregulated in KPPC tumors compared to normal liver (Figure 2F).

### Pan-LOX inhibition reduces IP and increases blood vessel patency, which is associated with enhanced chemotherapeutic efficacy in an orthotopic model of iCCA

To evaluate novel therapeutic strategies for treating iCCA, we previously generated the syngeneic murine cell line URCCA4.3 derived from a spontaneously occurring KPPC iCCA tumor.^25^ URCCA4.3 cells orthotopically implanted in the liver develop tumors that faithfully recapitulate the dense stroma of autochthonous iCCA (Supplemental Figure S7A, http://links.lww.com/HC9/A993). Furthermore, URCCA4.3 tumors are prominently infiltrated with leukocytes and myeloid cells, including monocytic myeloid derived suppressor cell, granulocytic myeloid-derived suppressor cell, and TAMs, reproducing the immunosuppressive tumor immune microenvironment of CCA (Supplemental Figure S7B, C, http://links.lww.com/HC9/A993).

**FIGURE 3:**
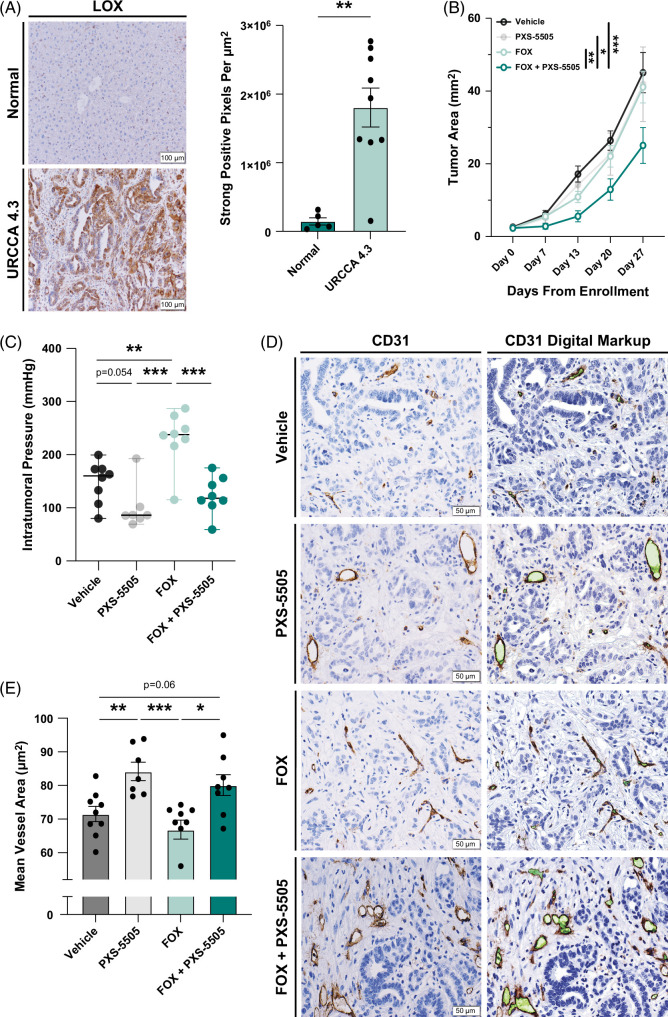
Pan-lysyl oxidase inhibition reverses intratumoral pressure, resulting in increased tumor vessel patency in intrahepatic cholangiocarcinoma. (A) Representative images compare immunohistochemistry staining of LOX isoforms in normal C57BL/6 livers versus orthotopic URCCA4.3 tumors at the end of life. End of life was defined as mice that appeared clinically moribund (body condition score <2) and/or those with gross ascites. The graph compares the quantification of LOX immunohistochemistry staining in normal C57BL/6 livers (n=5) versus orthotopic URCCA4.3 tumors (n=9) at the end of life. Data points represent whole-section quantification of LOX isoform staining per tissue specimen. (B) After confirmation of disease onset by ultrasonography 2 weeks following implantation, URCCA4.3 tumor-bearing mice were enrolled into indicated treatment cohorts. The graph compares tumor growth by ultrasonography measurement of the largest cross-sectional tumor area over time of orthotopic URCCA4.3 tumors treated as indicated. *p*-value determined by linear regression. n=9–10. (C) Graph shows intratumoral pressure measurements in orthotopic URCCA4.3 tumors after 4 weeks of treatment as indicated. n=7–8. Data points represent the mean of 3 pressure measurements obtained from separate regions of each tumor in vivo. (D) Representative images compare CD31 immunohistochemistry staining and associated digital markups used for quantification in tissue sections from orthotopic URCCA4.3 tumors treated for 4 weeks as indicated. Images were acquired at 200x magnification. (E) Graph compares mean vessel area in intrahepatic cholangiocarcinoma tumors using the Aperio Microvessel Algorithm after orthotopic URCCA4.3 tumors were treated for 4 weeks as indicated. n=7–8. Data points represent whole-section quantification of mean vessel area per tumor specimen. Abbreviations: FOX, fluorouracil plus oxaliplatin; LOX, lysyl oxidases.

To verify LOX expression in our iCCA orthotopic model, we performed IHC analysis with URCCA4.3 tumors, which also demonstrated significantly elevated protein levels that mirrored the expression patterns observed in the malignant epithelium and tumor stroma of KPPC iCCA tumors (Figure 3A, Supplemental Figure S8A, http://links.lww.com/HC9/A993). Since all 5 LOX isoforms were elevated in human and murine iCCA, we sought to assess the impact of pan-LOX inhibition on iCCA tumorigenesis with the irreversible small molecule pan-LOX inhibitor PXS-5505 in URCCA4.3 tumor-bearing mice, which achieved robust plasma levels and target engagement when administered in the diet (Supplemental methods, supplemental Figure S8B, C, http://links.lww.com/HC9/A993). To evaluate the potential of pan-LOX inhibition for improving the efficacy of standard therapy for treating iCCA, URCCA4.3 tumor-bearing mice were randomized into cohorts of vehicle, PXS-5505, and weekly doses of FOX with or without PXS-5505. While treatment with PXS-5505 or FOX alone had no impact on tumor growth relative to the vehicle, the combination of PXS-5505 with FOX significantly delayed tumor growth compared to all other cohorts, strongly suggesting pan-LOX inhibition abrogates resistance to chemotherapy (Figure 3B).

Tumor-induced mechanical compression associated with vascular collapse and poor delivery of chemotherapy has been described in cancers with prominent desmoplasia.^28^^,^^29^ Therefore, we investigated whether pan-LOX inhibition with PXS-5505 could reverse intratumoral compression and enhance vascular perfusion in iCCA tumors. To assess changes in mechanical compression in response to pan-LOX inhibition, we measured IP prior to explant using a pressure catheter probe in URCCA4.3 tumor-bearing mice following 4 weeks of treatment with vehicle, PXS-5505, and FOX with or without PXS-5505. Interestingly, tumors treated with FOX alone demonstrated significantly increased IP relative to vehicle, suggesting chemotherapy exacerbated vascular collapse, a potential adverse effect of cytotoxic drugs limiting therapeutic efficacy (Figure 3C). In contrast, tumors treated with PXS-5505 alone or in combination with FOX exhibited decreased IP compared to both vehicle and FOX, respectively. Utilizing a novel in-house assay,^30^ we observed a significant reduction in tumor LOXL2 activity in PXS-5505-treated cohorts, providing evidence of sustained drug delivery and target engagement (Supplemental methods, supplemental Figure S9, http://links.lww.com/HC9/A993). Consistent with reductions in IP, analysis of IHC staining for CD31 revealed that PXS-5505-treated tumors had significantly increased mean blood vessel area compared to tumors treated with vehicle or FOX alone, demonstrating that pan-LOX inhibition also increased blood vessel patency (Figure 3D, E).

### Pan-LOX inhibition improves chemotherapeutic delivery and enhances cytotoxicity

**FIGURE 4:**
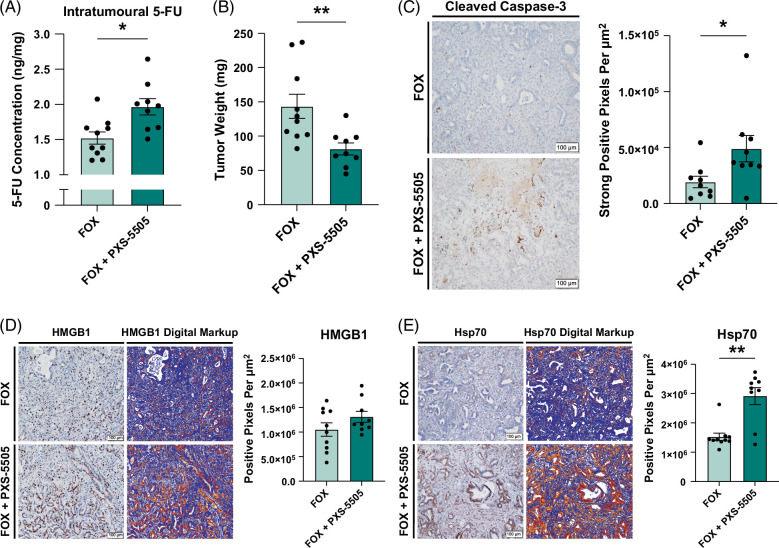
Pan-lysyl oxidase inhibition enhances chemotherapeutic penetration to facilitate cytotoxic and immunogenic cell death in intrahepatic cholangiocarcinoma tumors. (A) Graph shows the quantification of intratumoral 5-FU by UPLC-MS/MS in mice treated for 4 weeks as indicated. n=9–10 mice per group. Data points represent quantification of 5-FU (ng/mg) per tumor specimen. (B) Graph compares tumor weight of orthotopic URCCA4.3 tumors after 4 weeks of treatment as indicated. n=9–10 mice per group. Data points represent the weight (mg) of each individual tumor specimen (C–E) Representative images and graphs compare CC-3 (C), HMGB1 (D), and HSP70 (E) IHC staining and associated digital markups used for quantification in tissue sections from orthotopic URCCA4.3 tumors treated for 4 weeks as indicated. n=8–9 mice per group. Data points represent whole-section quantification of IHC staining per tumor specimen. Bar graphs depict mean ±SEM, and *p*-values were determined by Mann-Whitney *U* test. * = *p*<0.05, ** = *p*<0.01, *** = *p*<0.001, and **** = *p*<0.0001. Abbreviations: FOX, fluorouracil plus oxaliplatin; 5-FU, 5-fluorouracil; HMGB1, high-mobility group box 1; UPLC-MS/MS, ultra-performance liquid chromatography-mass spectrometry.

Pan-LOX inhibition with β-aminopropionitrile has been shown to increase intratumoral blood vessel patency and improve penetration of chemotherapy following exogenous LOXL2 administration.^31^ Since we observed that pan-LOX inhibition with PXS-5505 increased blood vessel patency in iCCA tumors, we hypothesized the efficacy of combination therapy was due to better perfusion of cytotoxic drug into tumors. Indeed, ultra-performance liquid chromatography-mass spectrometry analysis of explanted URCCA4.3 tumors treated with PXS-5505 in combination with FOX contained significantly higher levels of 5-FU compared to tumors that received FOX alone (Figure 4A). Importantly, URCCA4.3 tumors treated with combination therapy also weighed significantly less than tumors treated with chemotherapy alone (Figure 4B).

To examine underlying factors promoting chemotherapeutic efficacy in response to pan-LOX inhibition, we performed IHC analysis for markers associated with chemotherapy-induced cell death in URCCA4.3 tumors after treatment with FOX alone or in combination with PXS-5505. Notably, IHC analysis demonstrated that tumors treated with FOX plus PXS-5505 had significantly higher levels of cleaved caspase-3 staining, indicating that cells were undergoing increased apoptosis in response to chemotherapy (Figure 4C, Supplemental Figure S10, http://links.lww.com/HC9/A993). Furthermore, multiple damage-associated molecular patterns that mediate immunogenic cell death, including high-mobility group box 1 and heat shock protein 70 were elevated in tumors treated with combination therapy, suggesting that increased cytotoxicity induced by pan-LOX inhibition could potentiate antitumor immune responses (Figure 4D, E). Taken together, these data strongly suggest that pan-LOX inhibition with PXS-5505 promotes more efficient chemotherapy penetration, resulting in decreased tumor burden.

### Pan-LOX inhibition reduces fibrosis and reverses the balance in pro-tumor versus antitumor immune cell populations in iCCA tumors

**FIGURE 5:**
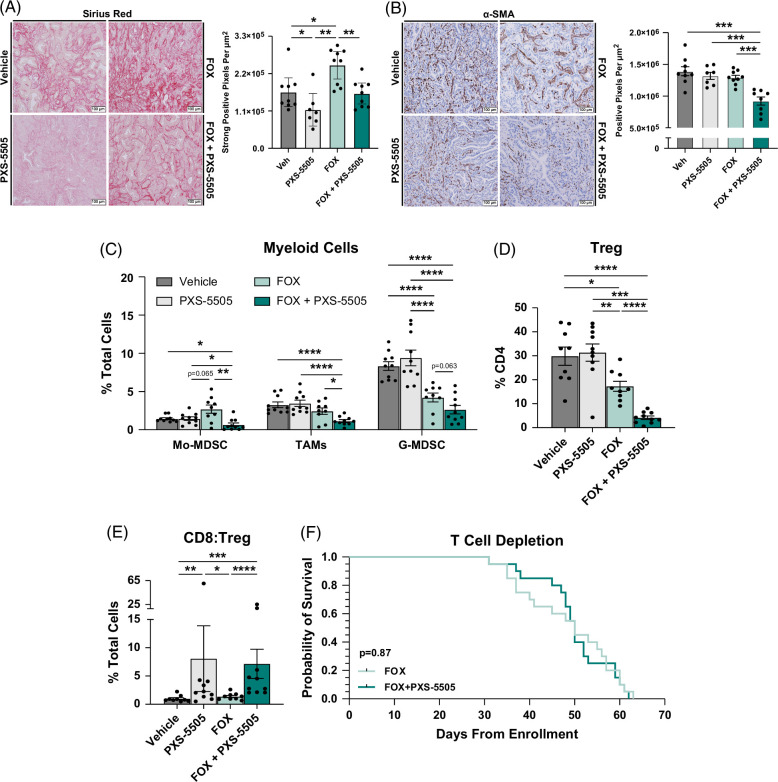
Pan-lysyl oxidase inhibition in combination with chemotherapy decreases fibrosis and reduces immunosuppression in the intrahepatic cholangiocarcinoma tumor microenvironment. (A) Representative images compare the degree of collagen deposition in orthotopic intrahepatic cholangiocarcinoma tumors by sirius red staining after mice were treated for 4 weeks as indicated. Graph shows the quantification of sirius red staining in tumors treated as indicated. n=8–9 mice per group. Data points represent whole-section quantification of sirius red staining per tumor specimen. (B) Representative images compare the prevalence of activated fibroblasts by α-SMA IHC staining in tissue sections from orthotopic URCCA4.3 tumors treated for 4 weeks as indicated. Graph shows the quantification of α-SMA IHC staining in tumors treated as indicated. n=8–9 mice per group. Data points represent whole-section quantification of α-SMA IHC staining per tumor specimen. (C–E) Graphs compare the prevalence of immunosuppressive myeloid subsets (C), CD4+FOXP3+ Treg cells (D), and the ratio of CD8:Treg (E) by flow cytometry analysis of orthotopic URCCA4.3 tumors after 3 weeks of treatment as indicated. n=9–10 mice per group. Data points represent the relative prevalence of the indicated cell type(s) per tumor specimen. (F) Kaplan-Meier curve compares the overall survival of mice with orthotopic intrahepatic cholangiocarcinoma; tumors enrolled into treatment groups as indicated and depleted of T cells. For T-cell depletion, mice were dosed i.p. with 500 μg of anti-CD8 (clone 2.43) and anti-CD4 (clone GK1.5) neutralizing IgG antibodies (BioXcell) prior to tumor implantation followed by 250 μg of each antibody every 4–5 days. *p*-value determined by log-rank (Mantel-Cox) test. Bar graphs depict mean ±SEM and *p*-values were determined by Mann-Whitney *U* test. * = *p*<0.05, ** = *p*<0.01, *** = *p*<0.001, and **** = *p*<0.0001. Abbreviations: FOX, fluorouracil plus oxaliplatin; G-MDSC, granulocytic myeloid-derived suppressor cell; Mo-MDSC, monocytic myeloid derived suppressor cell; α-SMA, alpha-smooth muscle actin; TAM, tumor-associated macrophage; Treg, T-regulatory.

LOXs facilitate collagen fibril cross-linking, a key pathway influencing the mechanical and cellular properties of a variety of stroma-rich tumors.^17^^–^^20^ Thus, we sought to examine iCCA tumors for changes in stromal composition in response to pan-LOX inhibition. Interestingly, quantification of sirius red staining showed that URCCA4.3 tumors treated with FOX alone had significantly higher levels of collagen deposition compared to vehicle suggesting that chemotherapy intensifies fibrosis, a possible mechanism of therapeutic resistance underlying the generation of greater mechanical force, which is in line with the increased IP we previously observed (Figure 5A, Supplemental Figure S11A, http://links.lww.com/HC9/A993). In contrast, treatment with PXS-5505 alone significantly reduced collagen deposition compared to vehicle and reversed chemotherapy-induced fibrosis. Notably, IHC analysis showed significant reductions in α-SMA in URCCA4.3 tumors treated with FOX plus PXS-5505 compared to all other treatment cohorts, suggesting the enhanced efficacy observed with combination therapy is also associated with loss of activated fibroblasts (Figure 5B, Supplemental Figure S11B, http://links.lww.com/HC9/A993). Taken together, these findings suggest pan-LOX inhibition directly impacts collagen deposition, but this is not solely mediated through activated fibroblasts. Instead, activated fibroblasts appear to be sensitized to chemotherapy-induced cell death and warrant further investigation.

Since damage-associated molecular patterns associated with immunogenic cell death CD were significantly elevated in URCCA4.3 tumors treated with combination therapy, we also investigated the dynamics of the tumor immune microenvironment in response to pan-LOX inhibition. Flow cytometry analysis of monocytic-derived myeloid cell subsets showed significant reductions in monocytic myeloid derived suppressor cells and TAMs in URCCA4.3 tumors treated with combination therapy versus all other treatment cohorts (Figure 5C, Supplemental Figure S12, http://links.lww.com/HC9/A993). In addition, the prevalence of TAM subsets expressing markers functionally associated with immunosuppression, including arginase 1 and programmed death-ligand 1 were also markedly lower in tumors treated with FOX plus PXS-5505 (Supplemental Figure S13A–C, http://links.lww.com/HC9/A993). Unlike monocytic subsets, the granulocytic fraction demonstrated more sensitivity to chemotherapy. In FOX-treated cohorts, granulocytic myeloid-derived suppressor cell significantly decreased compared to vehicle and PXS-5505-treated cohorts and trended even lower with combination therapy (Figure 5C, Supplemental Figure S12, http://links.lww.com/HC9/A993).

Given our observation of reduced myeloid cell populations, we assessed whether increased immunogenic cell death markers were associated with changes in tumor-infiltrating T-cell populations. Flow cytometry analysis of T-cell subsets revealed significant reductions in T-regulatory (Treg) cells with combination therapy compared to all other treatment cohorts (Figure 5D, Supplemental Figure S14A, http://links.lww.com/HC9/A993), resulting in a shift in the balance of CD4+ T-cell populations favoring T-helper function. Overall, reductions in Tregs corresponded with a significant increase in the ratio of CD8:Treg (Figure 5E, Supplemental Figure S14A, B, http://links.lww.com/HC9/A993), indicating the equilibrium between pro-tumor and antitumor immunity had been altered. Interestingly, neutralization of CD8 and CD4 T cells reversed the tumor control and survival advantage achieved with combination therapy, suggesting the shift in the CD8:Treg ratio was biologically important (Figure 5F, Supplemental Figure S14C, D, http://links.lww.com/HC9/A993).

### Pan-LOX inhibition disrupts macrophage invasiveness in iCCA

**FIGURE 6:**
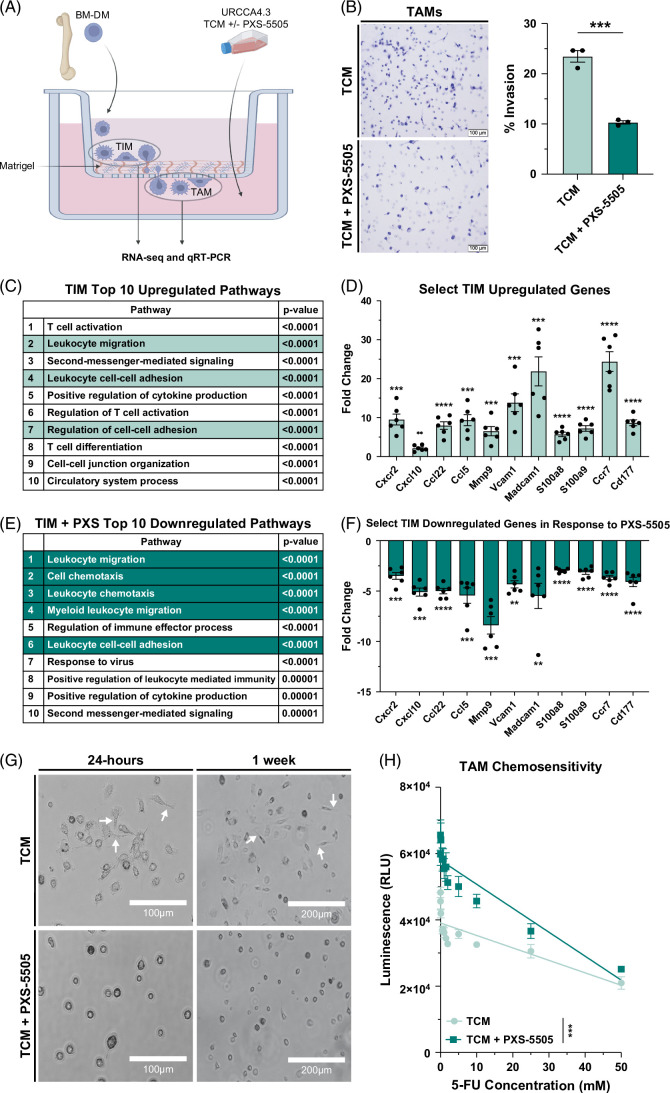
Pan-lysyl oxidase inhibition disrupts macrophage invasive characteristics and renders TAMs more susceptible to cytotoxic chemotherapy. (A) Schematic summarizes experimental design to evaluate the role of pan-LOX inhibition in CCA-induced macrophage trafficking into tumors. URCCA4.3 TCM was generated by filter-sterilizing CM that was seeded with cells at 25% confluency and incubated for 72 hours. (B) Representative images show the prevalence of TAM that traversed across Matrigel Invasion Chamber membranes in response to TCM or TCM plus PXS-5505 gradients. Images acquired at ×200. The graph compares the frequency of TAM that trafficked through Matrigel Invasion Chamber membranes in response to TCM (n=3) or TCM + PXS-5505 (n=3). Data points represent the percent of invasive TAMs per matrigel insert area. (C) Table identifies the top 10 upregulated GO pathways in TIM compared to TAM by ORA analysis. (D) Graph shows fold increase in select DEGs by quantitative real-time PCR analysis in upregulated GO pathways highlighted in red. Data points represent the mean fold increase of select TIM DEGs run in triplicate. (E) Table identifies the top 10 downregulated GO pathways in TIM + PXS-5505 compared to TIM by ORA analysis. (F) Graph shows fold decrease in select downregulated DEGs by quantitative real-time PCR analysis in downregulated GO pathways highlighted in blue. Data points represent the mean fold decrease of select TIM + PXS- 5505 DEGs run in triplicate. (G) Representative images show morphology of TAM cultured in TCM or TCM + PXS-5505 for 24 hours and 1 week. Arrows indicate TAMs with varying morphology characteristics. Images were acquired at ×100 and ×200 magnification. (H) Graph compares the viability of TAM after culture in TCM with or without PXS-5505 to increasing concentrations of chemotherapy. Data points depict mean ±SEM and the *p*-value was determined by simple linear regression. Bar graphs depict mean ±SEM with data points representing whole-section quantification of IHC staining per tumor specimen. *p-*values were determined by Mann-Whitney *U* test. ** = *p*<0.01, *** = *p*<0.001, and **** = *p*<0.0001. Abbreviations: BM-DM, bone-marrow-derived macrophages; RLU, relative light unit; TAM, tumor-associated macrophage; TCM, tumor-conditioned medium; TIM, tumor-infiltrating macrophage.

While we did not observe differences in TAM frequency in response to PXS-5505 alone, previous work has identified intricate relationships between stromal and tumor cell–directed LOXs with TAM functionality, including serving as a potent stimulus affecting macrophage invasive characteristics within glioblastoma.^16^^,^^32^^,^^33^ Furthermore, combination therapy greatly reduced TAMs, suggesting that PXS-5505 had impaired macrophage functionality and increased their sensitivity to cytotoxic therapy. We therefore sought to determine the effects of pan-LOX inhibition on macrophage function in iCCA tumors. To do so, we assessed the invasive potential of BM-DM by quantifying the number of tumor-infiltrating macrophages (TIMs) to traverse across Matrigel Invasion Chamber membranes to become TAMs in response to a URCCA4.3 TCM gradient supplemented with or without PXS-5505 (Figure 6A). Quantitative analysis of TAMs demonstrated that significantly less BM-DM migrated toward TCM supplemented with PXS-5505 compared to TCM alone, providing evidence that pan-LOX inhibition did indeed disrupt the invasive potential of TIMs (Figure 6B).

To further evaluate functional differences in the migratory properties of TIMs in response to pan-LOX inhibition, we isolated RNA from TIMs and TAMs cultured in URCCA4.3 TCM gradients with or without PXS-5505 and performed RNA-seq analysis. Principle component analysis of the 1470 DEGs showed that TIM and TAM were genomically distinct (Supplemental Table S7, http://links.lww.com/HC9/A994, Supplemental Figure S15, http://links.lww.com/HC9/A993). Over-representation analysis of the 1136 upregulated DEGs in TIM versus TAM demonstrated that among the top 10 significantly changed gene ontology pathways were processes associated with T-cell activation, leukocyte migration, leukocyte cell-cell adhesion, positive regulation of cytokine production, and regulation of cell-cell adhesion (Figure 6C). TaqMan qRT-PCR analysis validated the significant upregulation of several select genes that overlapped in pathways associated with migration and adhesion, including *Cxcr2*, *Cxcl10*, *Ccl22*, *Ccl5*, *Mmp9*, *Vcam1*, *Madcam1*, *S100a8*, *S100a9*, *Ccr7*, and *Cd177* (Figure 6D). In contrast, differential gene expression analysis of TIM cultured in TCM versus TIM cultured in TCM supplemented with PXS-5505 demonstrated significant changes in only 615 DEGs, but the majority were downregulated (Supplemental Table S7, http://links.lww.com/HC9/A994). Notably, over-representation analysis of the 550 downregulated DEGs showed that among the top 10 significantly changed gene ontology pathways were biological processes associated with leukocyte migration, leukocyte chemotaxis, myeloid leukocyte migration, leukocyte cell-cell adhesion, and positive regulation of cytokine production (Figure 6E). Moreover, every upregulated gene associated with migration and adhesion previously validated by Taqman qRT-PCR analysis was now significantly downregulated (Figure 6F), strongly suggesting that pan-LOX disrupts the migratory fitness of TIMs.

Interestingly, BM-DM cultured in TCM versus TCM supplemented with PXS-5505 appeared morphologically distinct (Figure 6G). BM-DM cultured in TCM transformed into elongated ameboid/mesenchymal shapes with cellular protrusions within 24 hours, a geometry more consistent with a migratory phenotype. In contrast, BM-DM cultured in TCM supplemented with PXS-5505 maintained a rounded shape for at least 7 days, an architecture much less consistent with migration. Importantly, PXS-5505 had no impact on BM-DM viability (Supplemental Figure S16, http://links.lww.com/HC9/A993).

### Pan-LOX inhibition sensitizes TAMs to chemotherapy-induced cell death

We observed that combination therapy with PXS-5505 plus FOX was the only treatment arm that significantly reduced orthotopic tumor burden and decreased the frequency of tumor-associated monocytic lineages in vivo. Thus, we sought to determine if the deficits in migratory pathways induced by pan-LOX inhibition were linked to monocytic myeloid derived suppressor cells/TAMs sensitivity to chemotherapy. To evaluate the sensitivity of tumor-associated monocytic lineages to chemotherapy in response to pan-LOX inhibition, we cultured TAMs in TCM with or without PXS-5505 in wells coated with basement membrane extract for 24 hours and then exposed them to varying concentrations of 5-FU for an additional 24 hours. CellTiter-Glo analysis demonstrated that TAMs cultured in TCM with PXS-5505 were significantly less viable after treatment with 5-FU compared to TAMs cultured in TCM alone, demonstrating that pan-LOX inhibition sensitized TAMs to chemotherapy (Figure 6H). This synergistic effect was not observed with URCCA4.3 tumor cells in vitro, suggesting the benefits of PXS-5505 were dependent on alterations in the TME of iCCA rather than direct cytotoxicity of tumor cells (Supplemental Figure S17, http://links.lww.com/HC9/A993).

### Combination therapy with pan-LOX inhibition restrains KPPC murine iCCA

**FIGURE 7:**
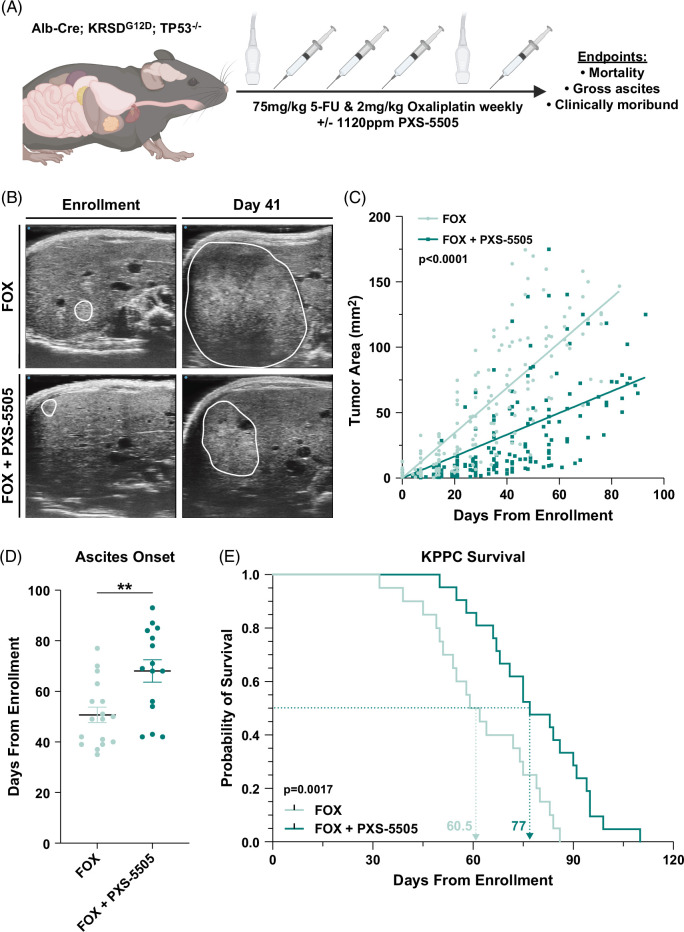
Combining chemotherapy with pan-lysyl oxidase inhibition decreases tumor progression, slows onset of ascites, and significantly increases survival in the KPPC mouse model of intrahepatic cholangiocarcinoma. (A) Schematic outlines the experimental design for KPPC mice serially enrolled into treatment regimens as indicated. After confirmation of disease onset by ultrasound, KPPC mice were started on a therapeutic trial of FOX (75 mg/kg 5-fluorouracil plus 2 mg/kg oxaliplatin, i.p. weekly) and nutritionally complete chow (Research Diets) formulated with or without the small-molecule pan-LOX inhibitor PXS-5505 (Pharmaxis Ltd) at 1120 ppm. iCCA histopathology was confirmed postmortem in all available KPPC tumors by a board-certified pathologist. (B) Representative sonographs of the largest tumor cross-sectional areas show sizes of tumors at time of enrollment (day 0) and while mice are on therapy (day 41) in treatment groups as indicated. (C) Graph compares tumor size progression over time by US measurement of the largest cross-sectional area of the tumor in KPPC mice treated with FOX (n=20) or FOX combined with PXS-5505 (n=21). Each data point represents individual tumor assessments over time. *p*-value determined by linear regression. (D) Graph compares onset of ascites determined by the US in KPPC mice treated as indicated. Each data point represents individual KPPC mice at time of ascites onset. Graph depicts mean ±SEM and p-value determined by Mann-Whitney *U* test. ** = *p*<0.01. (E) Kaplan-Meier survival curve compares the overall survival of KPPC mice enrolled into indicated treatment cohorts at onset of disease. Dashed arrows indicate median overall survival for mice in FOX (red) versus FOX combined with PXS-5505 (blue) cohorts. The *p*-value was determined by log-rank (Mantel-Cox) test. Abbreviations: BM-DM, bone-marrow-derived macrophages; FOX, fluorouracil plus oxaliplatin; KPPC, LSL-Kras^G12D^; Tp53^Flox/Flox^; Alb-Cre.

KPPC iCCA tumors express high levels of all lysyl oxidase isoforms and pan-LOX inhibition with PXS-5505 increased the efficacy of chemotherapy in our orthotopic murine model of iCCA. Thus, we sought to test the effectiveness of pan-LOX inhibition with chemotherapy in the KPPC model of iCCA. Accordingly, KPPC mice were screened for disease onset with US and were serially enrolled into a therapeutic trial of weekly FOX with or without PXS-5505 (Figure 7A, B). At the time of enrollment, KPPC mice had similar baseline demographic and tumor characteristics between treatment cohorts, and tumor growth and disease burden were monitored by US over time (Supplemental Table S8, http://links.lww.com/HC9/B15). US assessment of tumor growth demonstrated that KPPC mice treated with FOX plus PXS-5505 had significantly restrained tumor growth and onset of ascites compared to mice treated with FOX alone (Figure 7B–D). Notably, FOX plus PXS-5505 improved overall survival compared to FOX alone, demonstrating the effectiveness of combination therapy with pan-LOX inhibition for treating autochthonous iCCA tumors (Figure 7E). Thus, taken together, these data suggest combination therapy with pan-LOX inhibition is a promising strategy for treating iCCA.

## DISCUSSION

The prognosis for CCA remains poor with few effective treatment strategies, in part due to robust desmoplastic stroma supported by LOXs (LOX, LOXL1–4) that contribute to therapeutic resistance.^10^^,^^20^^,^^34^^–^^37^ Here, we demonstrated that LOX isoform expression in human CCA is prognostic and that all 5 isoforms were elevated in human and murine iCCA. pan-LOX inhibition with PXS-5505 in autochthonous and orthotopic murine models of iCCA demonstrated improved responses to chemotherapy and enhanced antitumor immunity.

Stroma-rich tumors contain a dense collagen framework that exerts tensile forces compressing structurally weak tumor vasculature, limiting chemotherapeutic penetration.^28^^,^^29^^,^^38^^,^^39^ LOXs mediate this stress through modification of collagen cross-linking, influencing both fiber width and orientation.^32^^,^^40^^–^^43^ LOXL2 has been clinically targeted; however, inhibition of this isoform alone failed to disrupt fibrosis, underscoring the potential contribution from additional LOX isoforms.^44^^,^^45^ As such, pan-LOX inhibition offers a promising strategy to combat chemotherapy resistance^17^ and improve penetration of systemic therapies for treating stroma-rich tumors.^31^^,^^43^ In fact, pan-LOX inhibition with PXS-5505 has recently been shown to alter the highly desmoplastic TME of the Kras^G12D/+^; Trp53^R172H/+^; P48-Cre pancreatic ductal adenocarcinoma model,^24^ rendering it susceptible to chemotherapy and providing insight into its potential therapeutic benefit. Similarly, we found that PXS-5505 disrupted the stromal framework of our iCCA murine model to enhance the efficacy of chemotherapy. Mechanistically, pan-LOX inhibition reversed chemotherapy-induced fibrosis, sustaining vascular patency and enhancing chemotherapeutic penetrance. Together, these findings suggest that modulation of tumor stroma through pan-LOX inhibition primes the TME for improved responses to systemic therapy.

TAMs have recently been shown to modulate the TME of stroma-rich tumors through upregulation of LOXs, promoting tumor progression and metastatic potential.^16^^,^^31^ This interplay between desmoplastic stroma and immune components of the TME facilitates immune evasion and treatment resistance in CCA.^46^ In particular, subtypes of CCA with predominant myeloid infiltrates portend worse prognosis than those with elevated CD8 and helper T-cell lineages.^47^ Our autochthonous and orthotopic murine models display a robust myeloid infiltrate that recapitulates the immunosuppressive TME of CCA, and we have previously shown that TAMs within this model are alternatively polarized toward an immunosuppressive (M2) phenotype that drives an immunologically cold microenvironment.^25^ Interestingly, while we found no differences with pan-LOX inhibition alone, combination therapy with PXS-5505 reduced myeloid immune suppression, which was associated with improved antitumor T-cell immunity. Through pan-LOX inhibition with PXS-5505, we impacted macrophage functionality by downregulating macrophage invasive signatures and rendering TAMs susceptible to cytotoxic cell death. These findings support the notion that tumor architecture contributes to therapeutic resistance by additionally limiting effective antitumor immune responses.^48^ LOXs contribute to the construct of this matrix, and pan-LOX inhibition offers an avenue to modify CCA and render it more susceptible to chemotherapy-induced antitumor responses. Future efforts should explore immune checkpoint inhibition in this context to augment this therapeutic impact.

Combined, these results highlight pan-LOX inhibition with PXS-5505 as an attractive therapeutic adjunct to combat treatment resistance in CCA. While β-aminopropionitrile has historically been used to study pan-LOX inhibition, it has off-target effects and does not lend itself to clinical application.^49^^,^^50^ In contrast, PXS-5505 represents a highly selective, orally dosed, small-molecule pan-LOX inhibitor that eliminates off-target effects due to nonspecific processing by other human amine oxidases and has demonstrated safety and tolerability in myelofibrosis (NCT04676529).^51^

## CONCLUSIONS

CCA tumors contain abundant LOX isoform expression that inversely correlates with patient outcomes. Pan-LOX inhibition with PXS-5505 reduces chemotherapy-induced mechanical compression of tumor vasculature to improve chemotherapeutic delivery and efficacy. Pan-LOX inhibition additionally reduces myeloid immune cell populations and is associated with improved antitumor T-cell responses. Thus, pan-LOX inhibition represents a novel strategy to augment current systemic therapeutics for treating CCA.

## Supplementary Material
