# Supplementary material for: Pan-lysyl oxidase inhibition disrupts fibroinflammatory tumor stroma, rendering cholangiocarcinoma susceptible to chemotherapy
Source: Hepatol Commun. 2024 Aug 5;8(8):e0502. doi: 10.1097/HC9.0000000000000502 (PMC11299993; doi:10.1097/HC9.0000000000000502)
Supplement: Supplementary file 1 [file hc9-8-e0502-s001.docx]

**Supplemental Table 1**

| **Antibody** | **Manufacturer** | **Host** | **Clone** | **Reactivity** | **Application** | **Dilution** |
| --- | --- | --- | --- | --- | --- | --- |
| α-SMA | Cell Signaling | Rabbit | D4K9N | Human, Mouse | IHC | 1:640 |
| LOX | Abcam | Rabbit | EPR4025 | Human, Mouse | IHC, IF | 1:900 |
| CD31 | Cell Signaling | Rabbit | D8V9E | Human, Mouse | IHC | 1:100 |
| CK7 | Bio SB | Mouse | OV-TL 12/30 | Human | IF | 1:100 |
| CC3 | Cell Signaling | Rabbit | D3E9 | Mouse | IHC | 1:250 |
| HMGB1 | Cell Signaling | Rabbit | D3E5 | Mouse | IHC | 1:800 |
| Hsp70 | Abcam | Mouse | 5A5 | Mouse | IHC | 1:200 |

**Supplemental Table 2**

| **Antibody** | **Manufacturer** | **Host** | **Clone** | **Reactivity** | **Application** | **Dilution** |
| --- | --- | --- | --- | --- | --- | --- |
| F4/80 | Thermo Fisher | Rat | CI:A3-1 | Mouse | FC | 1:10 |
| MHCII | Biolegend | Rat | M5/114.15. | Mouse | FC | 1:1000 |
| Ly6G | Biolegend | Rat | 1A8 | Mouse | FC | 1:50 |
| CD68 | Biolegend | Rat | FA-11 | Mouse | FC | 1:40 |
| CD11b | Biolegend | Rat | M1/70 | Mouse | FC | 1:80 |
| CD206 | Biolegend | Rat | C068C2 | Mouse | FC | 1:100 |
| Arg1 | ThermoFisher | Rat | A1exF5 | Mouse | FC | 3:1000 |
| CD45 | Biolegend | Rat | 30-F11 | Mouse | FC | 1:80 |
| PD-L1 | Biolegend | Rat | 10F.9G2 | Mouse | FC | 1:80 |
| Ly6C | Biolegend | Rat | HK1.4 | Mouse | FC | 1:200 |
| CD3e | Biolegend | Hamster | 145-2C11 | Mouse | FC | 1:50 |
| CD8a | Biolegend | Rat | 53-6.7 | Mouse | FC | 1:20 |
| CD49b | Biolegend | Rat | DX5 | Mouse | FC | 1:50 |
| CD19 | Biolegend | Rat | 6D5 | Mouse | FC | 1:40 |
| NK1.1 | Biolegend | Mouse | PK136 | Mouse | FC | 3:500 |
| Foxp3 | eBioscience | Rat | FJK-16s | Mouse | FC | 1:50 |
| CD4 | Santa Cruz | Rat | GK1.5 | Mouse | FC | 1:50 |
| CD45 | Biolegend | Rat | 30-F11 | Mouse | FC | 1:80 |
| CTLA-4 | Biolegend | Hamster | UC10-4B9 | Mouse | FC | 1:20 |
| PD-1 | Biolegend | Rat | RMP1-30 | Mouse | FC | 1:20 |

**Supplemental Table 3**

| **Gene Target** | **Manufacturer** | **Target Species** | **Assay ID** |
| --- | --- | --- | --- |
| LOX | ThermoFisher | Human | Hs00942483_m1 |
| LOXL1 | ThermoFisher | Human | Hs00935937_m1 |
| LOXL2 | ThermoFisher | Human | Hs01046941_g1 |
| LOXL3 | ThermoFisher | Human | Hs00158757_m1 |
| LOXL4 | ThermoFisher | Human | Hs00260059_m1 |
| LOX | ThermoFisher | Mouse | [Mm00495386_m1](https://www.thermofisher.com/taqman-gene-expression/product/Mm00495386_m1?CID=&ICID=&subtype=) |
| LOXL1 | ThermoFisher | Mouse | [Mm01145738_m1](https://www.thermofisher.com/taqman-gene-expression/product/Mm01145738_m1?CID=&ICID=&subtype=) |
| LOXL2 | ThermoFisher | Mouse | [Mm00804740_m1](https://www.thermofisher.com/taqman-gene-expression/product/Mm00804740_m1?CID=&ICID=&subtype=) |
| LOXL3 | ThermoFisher | Mouse | [Mm00442953_m1](https://www.thermofisher.com/taqman-gene-expression/product/Mm01184865_m1?CID=&ICID=&subtype=) |
| LOXL4 | ThermoFisher | Mouse | [Mm00446385_m1](https://www.thermofisher.com/taqman-gene-expression/product/Mm00446385_m1?CID=&ICID=&subtype=) |
| Cxcr2 | ThermoFisher | Mouse | Mm99999117_s1 |
| Cxcl10 | ThermoFisher | Mouse | Mm00445235_m1 |
| Ccl22 | ThermoFisher | Mouse | [Mm00436439_m1](https://www.thermofisher.com/taqman-gene-expression/product/Mm00436439_m1?CID=&ICID=&subtype=) |
| Ccl5 | ThermoFisher | Mouse | Mm01302427_m1 |
| Mmp9 | ThermoFisher | Mouse | Mm00442991_m1 |
| Vcam1 | ThermoFisher | Mouse | Mm01320970_m1 |
| Madcam1 | ThermoFisher | Mouse | Mm00522088_m1 |
| S100a8 | ThermoFisher | Mouse | Mm00496696_g1 |
| S100a9 | ThermoFisher | Mouse | Mm00656925_m1 |
| Ccr7 | ThermoFisher | Mouse | Mm99999130_s1 |
| Cd177 | ThermoFisher | Mouse | Mm00503537_m1 |

**Supplemental Table 4**

| **Characteristics** | **Resected CCA (n = 42)** |
| --- | --- |
| **Age (mean, SD)** | 61.2 (14.3) |
| **Sex**  Male  Female | 24 (57%)  18 (43%) |
| **Ethnicity**  White  African American  Non-White | 36 (86%)  5 (12%)  1 (2%) |
| **BMI (mean, SD)** | 27.60 (4.91) |
| **Etiology**  Sporadic  Primary Sclerosing Cholangitis  Hepatitis C  Cryptogenic Cirrhosis  Wilson’s Disease | 35 (83%)  3 (7%)  2 (5%)  1 (2%)  1 (2%) |
| **Anatomy of Lesion**  Intrahepatic  Perihilar  Extrahepatic | 26 (62%)  12 (29%)  10 (12%) |
| **Type of Resection**  Wedge  Bile Duct Resection  Segmentectomy  Lobectomy  Trisegmentectomy  Whipple  Transplantation | 2 (5%)  2 (5%)  11 (26%)  11 (26%)  5 (12%)  5 (12%)  6 (14%) |
| **T Stage**  T1  T2  T3  T4  Unknown | 12 (29%)  12 (29%)  11 (26%)  5 (12%)  2 (5%) |
| **N Stage**  N0  N1  N2  NX  Unknown | 12 (29%)  11 (26%)  0 (0%)  14 (33%)  5 (12%) |
| **Lymphovascular Invasion Present** | 17 (40%) |
| **Perineural Invasion Present** | 17 (40%) |
| **Histologic Grade**  G1  G2  G3 | 8 (19%)  22 (52%)  12 (29%) |
| **Margin Status**  R0  R1  R2 | 35 (83%)  5 (12%)  2 (5%) |

**Supplemental Table 5**

| **Parameter** | **Hazard** **Ratio** | **95% Hazard Ratio**  **Confidence Limits** | |
| --- | --- | --- | --- |
| High LOX expression  (Ref = Low LOX expression) | 2.147 | 0.836 | 5.518 |
| T stage 3 or 4  (Ref = 1 or 2) | 1.572 | 0.588 | 4.204 |
| Positive lymph nodes  (Ref = negative lymph nodes) | 2.965 | 0.832 | 10.564 |
| Unknown lymph nodes  (Ref = negative lymph nodes) | 2.073 | 0.648 | 6.626 |
| LVI present  (Ref = Not present) | 0.428 | 0.178 | 1.028 |
| Moderately Differentiated  (Ref = Well differentiated) | 1.806 | 0.437 | 7.464 |
| Poorly Differentiated  (Ref = Well differentiated) | 2.303 | 0.531 | 9.990 |

**Supplemental Table 6**

| **Parameter** | **Hazard** **Ratio** | **95% Hazard Ratio**  **Confidence Limits** | |
| --- | --- | --- | --- |
| High LOX expression  (Ref = Low LOX expression) | 3.285 | 0.965 | 11.186 |
| T stage 3 or 4  (Ref = 1 or 2) | 2.616 | 0.634 | 10.793 |
| Positive lymph nodes  (Ref = negative lymph nodes) | 4.015 | 0.769 | 20.967 |
| Unknown lymph nodes  (Ref = negative lymph nodes) | 3.369 | 0.606 | 18.737 |
| LVI present  (Ref = Not present) | 0.418 | 0.106 | 1.646 |
