# Supplementary material for: Pan-lysyl oxidase inhibition disrupts fibroinflammatory tumor stroma, rendering cholangiocarcinoma susceptible to chemotherapy
Source: Hepatol Commun. 2024 Aug 5;8(8):e0502. doi: 10.1097/HC9.0000000000000502 (PMC11299993; doi:10.1097/HC9.0000000000000502)
Supplement: Supplementary file 2 [file hc9-8-e0502-s002.pdf]

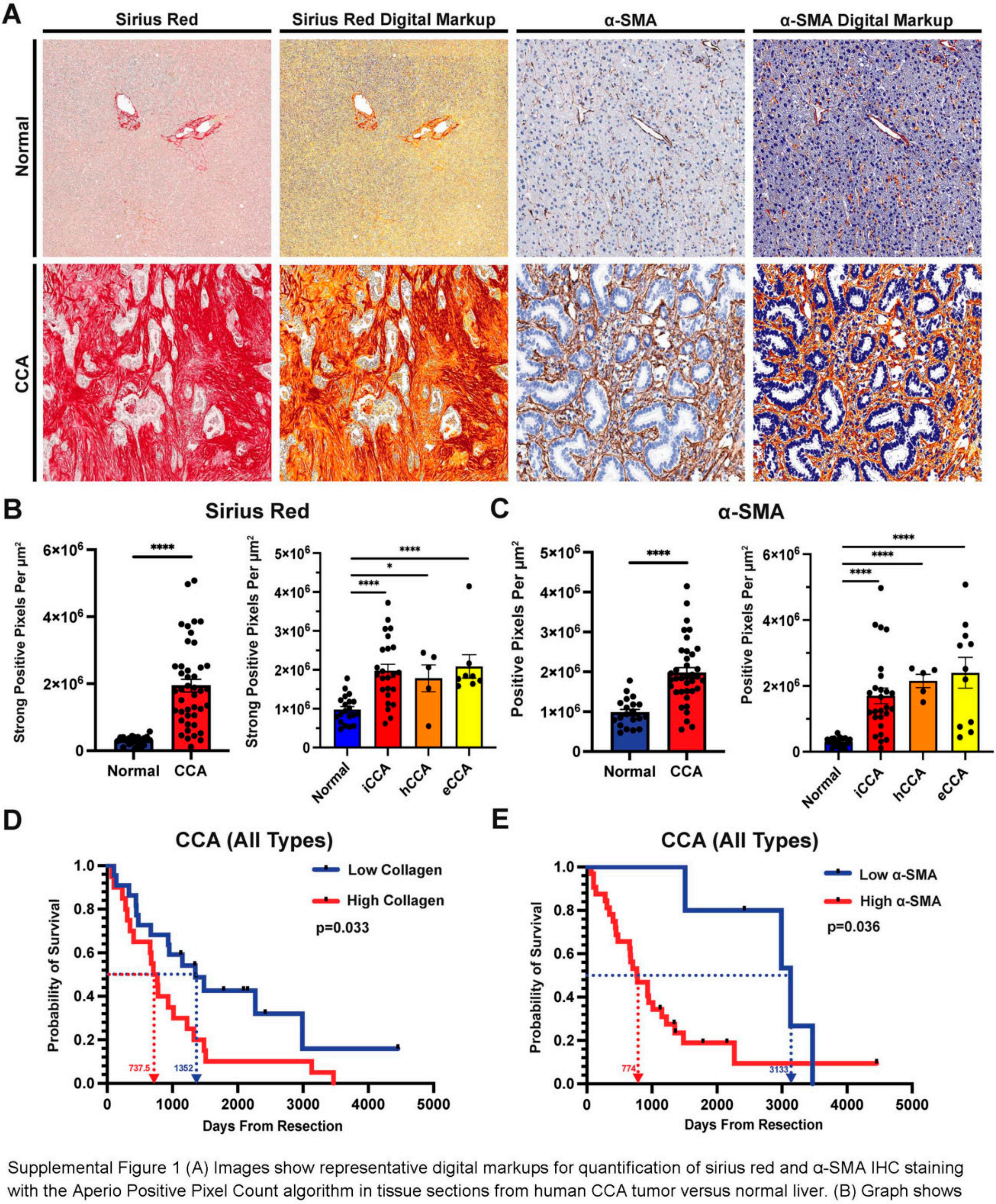

Supplemental Figure 1 (A) Images show representative digital markups for quantification of sirius red and α-SMA IHC staining with the Aperio Positive Pixel Count algorithm in tissue sections from human CCA tumor versus normal liver. (B) Graph shows the quantification of sirius red staining in normal liver specimens (n=20) and resected CCA tumors (n=43). Datapoints represent whole-section quantification of sirius red staining per tissue specimen. (C) Kaplan-Meier curve compares OS after tissue sections from resected CCA tumor specimens were stratified into low (n=22) versus high (n=21) sirius red staining cohorts. (D) Graph shows the quantification of sirius red staining in normal liver specimens (n=20) and CCA tumors stratified by subtype: intrahepatic (iCCA, n=26), hilar (hCCA, n=5), and extrahepatic (eCCA, n=11). Datapoints represent whole-section quantification of sirius red staining per tissue specimen. (E) Graph compares the quantification of α-SMA IHC staining in normal liver (n=20) versus CCA tumors (n=37). Datapoints represent whole-section quantification of α-SMA IHC staining per tissue specimen. (F) Kaplan-Meier curve compares OS after tissue sections from resected CCA tumor specimens were stratified into low (n=5) versus high (n=32) α-SMA staining cohorts. (G) Graph compares the quantification of α-SMA IHC staining in normal liver (n=20) versus CCA tumors stratified by subtype: intrahepatic (iCCA, n=24), hilar (hCCA, n=5), and extrahepatic (eCCA, n=8). Datapoints represent whole-section quantification of α-SMA IHC staining per tissue specimen. Bar graphs depict mean ±SEM and p-values determined by Mann-Whitney U test. * = p<0.05 and **** = p<0.0001 Dashed arrows indicate median OS for low (blue) versus high (red) staining cohorts and p-values for Kaplan-Meier determined by log-rank (Mantel-Cox) test with maximal significance cut-offs determined by the survminer package (The Comprehensive R Archive Network).

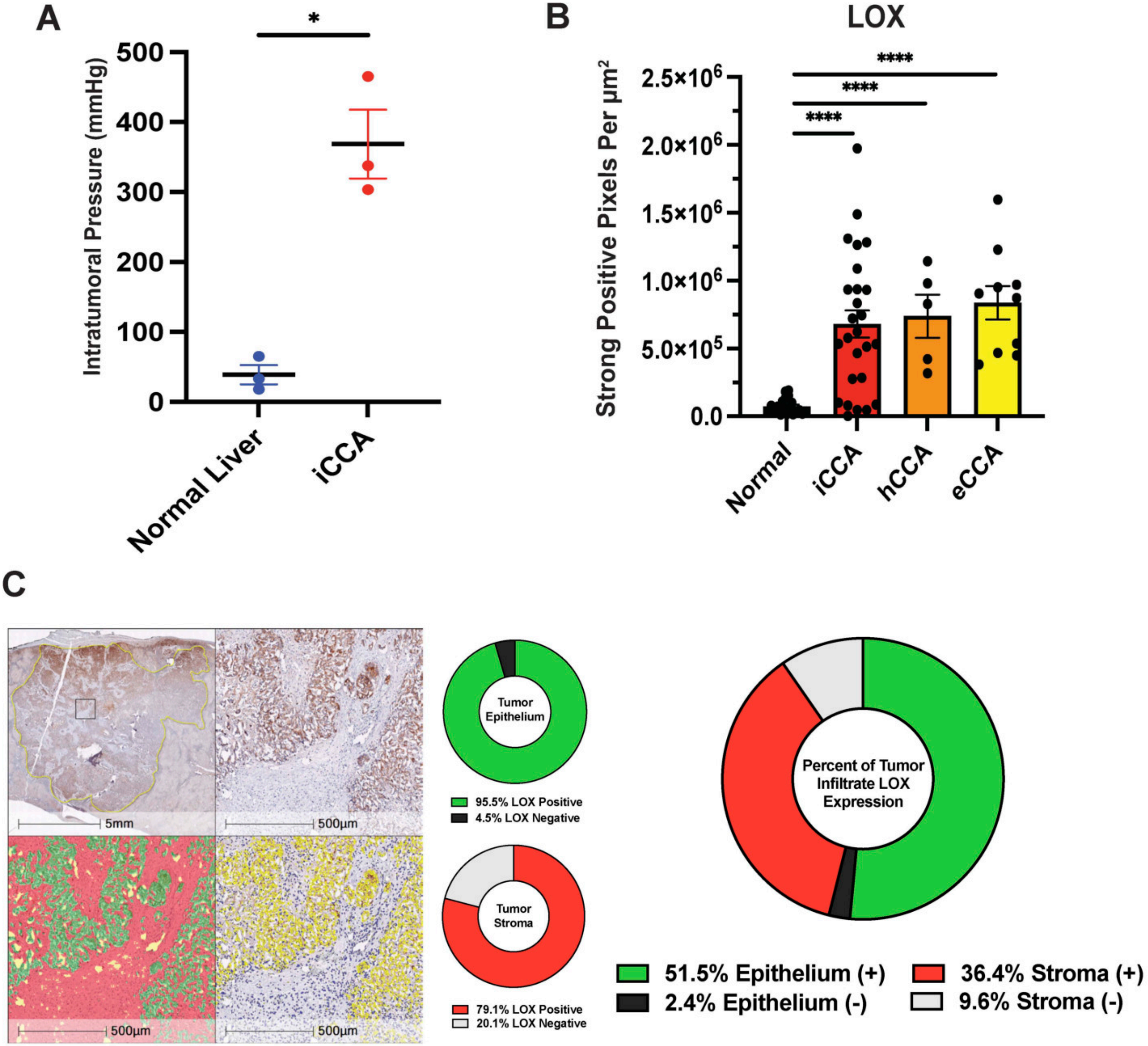

Supplemental Figure 2 (A) Graph shows measurements of intratumoral pressure in resected human intrahepatic CCA (iCCA) and nonadjacent normal liver. Intratumoral pressure was measured within 30 minutes of surgical resection using the Miller SPR-671 Mikro-Tip® pressure catheter, as previously described. Datapoints represent the mean of three pressure measurements obtained from separate regions of each tissue specimen. (B) Graph compares the quantification of LOX IHC staining in normal liver (n=20) versus CCA tumors stratified by subtype: intrahepatic (iCCA, n=26), hilar (hCCA, n=5), and extrahepatic (eCCA, n=10). Datapoints represent whole-section quantification of α-SMA IHC staining per tissue specimen. (C) Representative images show HALO analysis digital quantification of epithelial (green) and stromal (red) cell components within archival human CCA specimens previously stained for LOX with IHC. Representative image shown of Halo analysis digital quantification of LOX (yellow) expression per epithelial and stromal cell. Pie charts illustrate the prevalence of LOX-positive and LOX-negative cells per epithelial and stromal cell populations, as well as a percent of total tumor cell infiltrate as determined by Halo analysis.

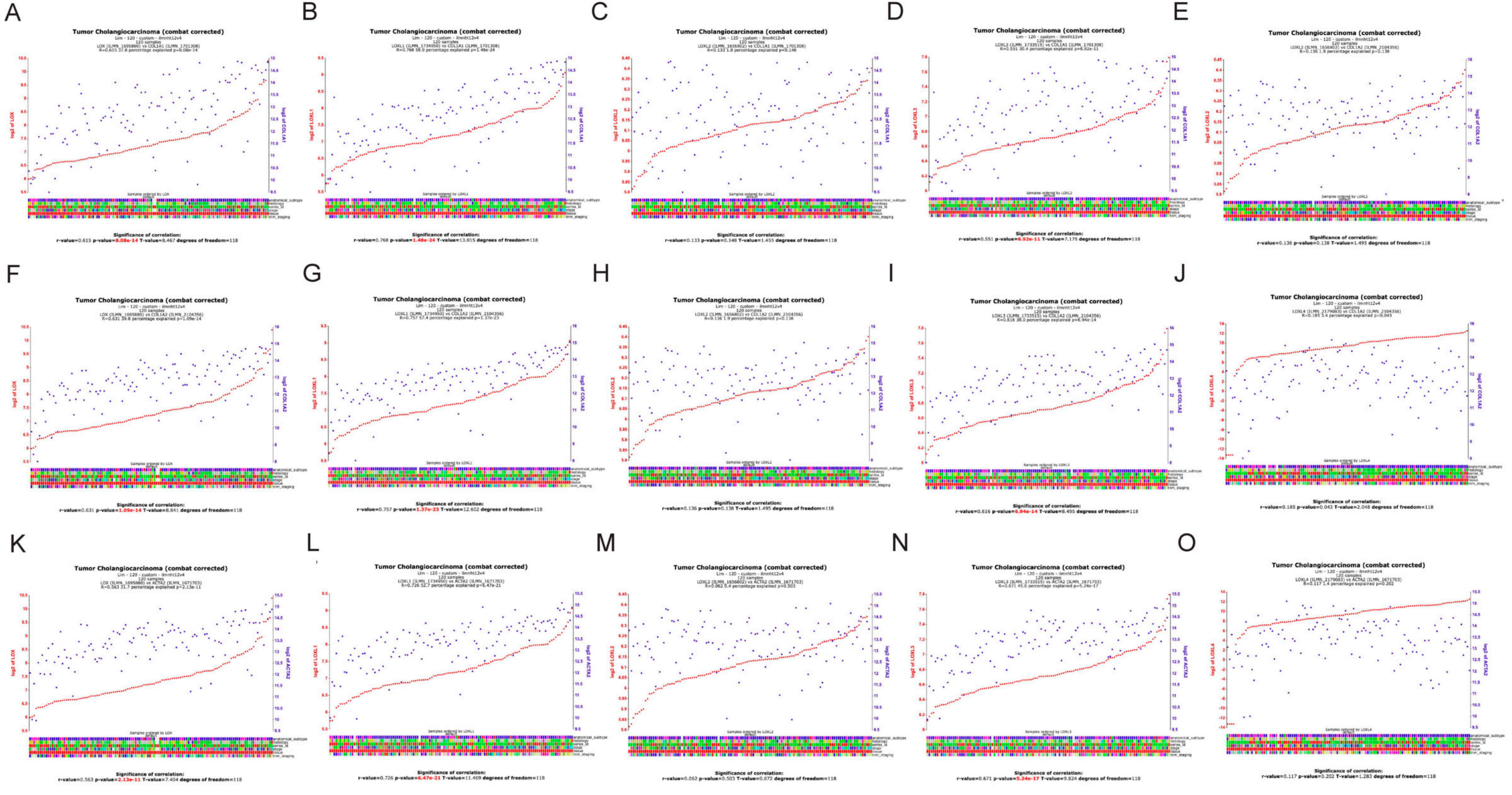

Supplemental Figure 3 Graphs show correlation of gene expression with the five lysyl oxidase isoforms and (A-E) COL1A1, (F-J) COL1A2, and (K-O) ACTA2 for a CCA dataset (n = 83). Data was obtained through the Lim dataset on the R2: Genomics Analysis and Visualization Platform.

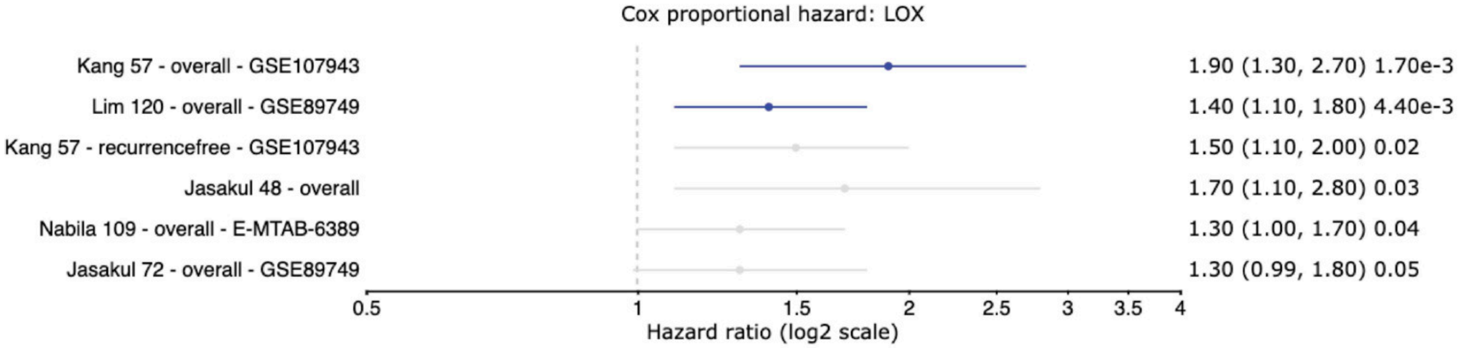

Supplemental Figure 4 Graph demonstrates cox proportional hazards regression of survival (overall and recurrence free, when appropriate) by LOX gene expression across CCA datasets within the R2: Genomics Analysis and Visualization Platform. Hazard ratio (HR) and significance depicted as: HR (95% confidence interval), p-value.

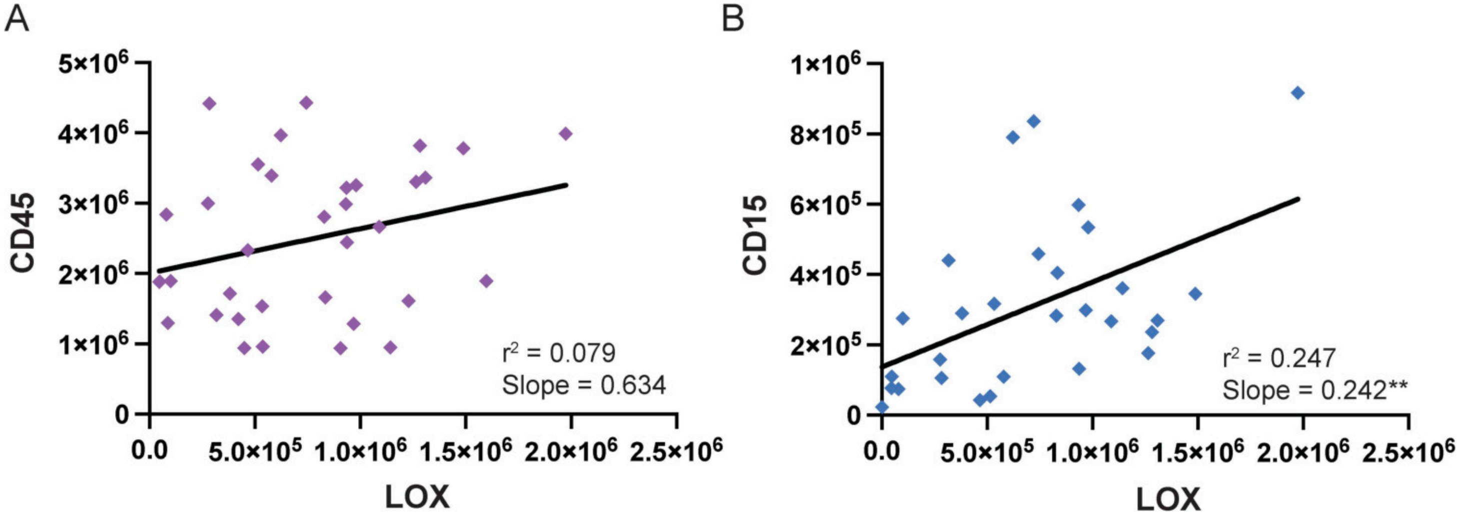

Supplemental Figure 5 Graphs show correlation of LOX expression with (A) CD45 (n=35) and (B) CD15 (n=29) IHC staining of archival human CCA tumors. Graphs depict correlation coefficient (r2) and best-fit slope as determined through simple linear regression. ** = $p < 0.01$ indicating significant deviation of slope from zero.

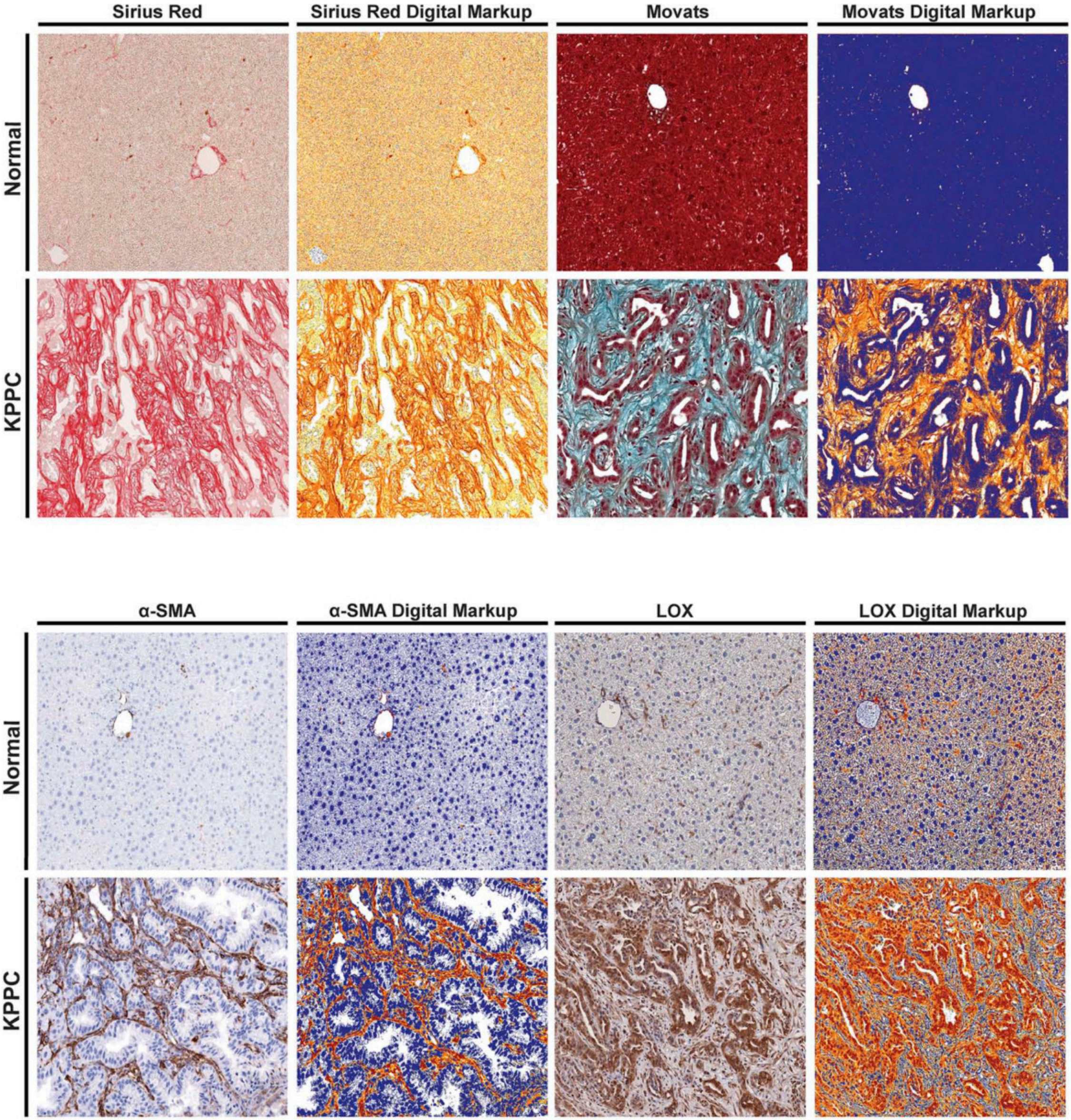

Supplemental Figure 6 Images demonstrate representative digital markups for quantification of sirius red, movat's pentachrome, α-SMA IHC, and LOX IHC staining with the Aperio Positive Pixel Count algorithm in tissue sections from normal livers of littermate controls and spontaneously occurring CCA tumors from KPPC mice at end of life. EOL was defined as mice that appeared clinically moribund (body condition score [BCS] <2) and/or those with gross ascites.

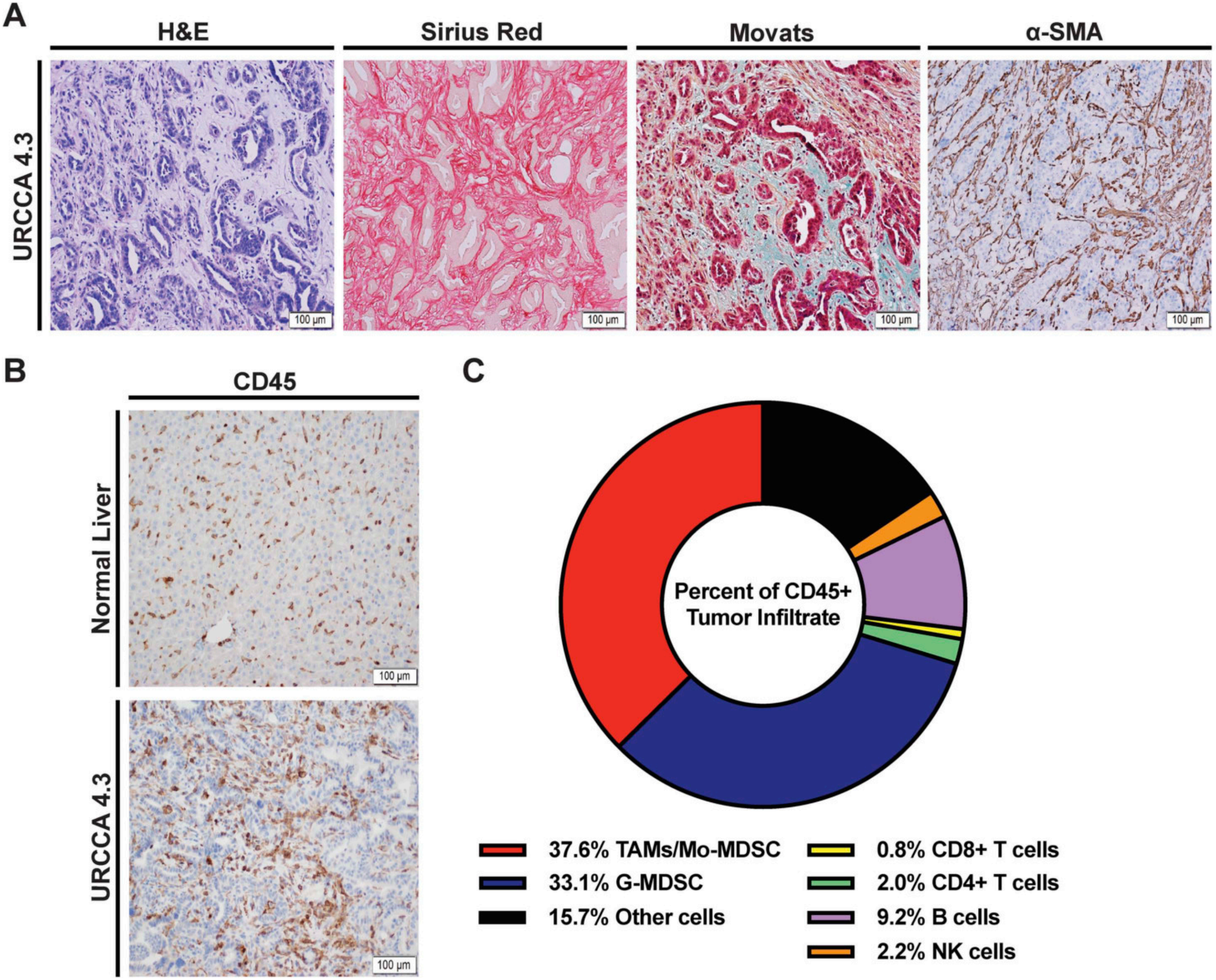

Supplemental Figure 7 (A) Representative images show H&E, sirius red, movat's pentachrome, and α-SMA IHC staining in tissue sections from mice with orthotopic URCCA4.3 CCA tumors at end of life. EOL was defined as mice that appeared clinically moribund (body condition score [BCS] <2) and/or those with gross ascites. Images were acquired at 200x magnification. (B) Representative images show CD45 IHC staining in tissue sections from normal livers of C57BL/6 mice versus mice with orthotopic URCCA4.3 CCA tumors at end of life. Images were acquired at 200x magnification. (C) Pie chart illustrates the prevalence of tumor-infiltrating immune cell subsets as a percent of CD45+ leukocytes as determined by flow cytometry analysis of orthotopic URCCA4.3 tumors. TAMs, tumor-associated macrophages; Mo-MDSC, monocytic myeloid-derived suppressor cells; G-MDSC, granulocytic myeloid-derived suppressor cells.

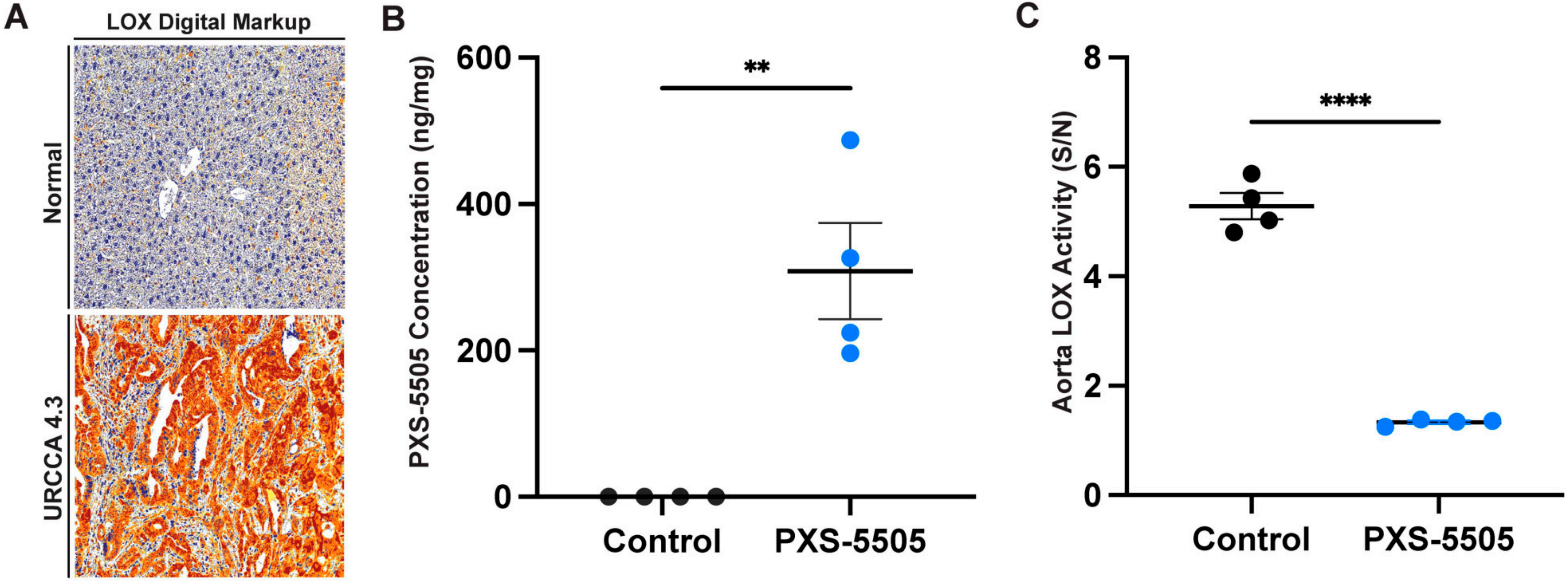

Supplemental Figure 8 (A) Images show representative digital markups for quantitation of LOX in tissue sections from normal liver of C57BL/6 mice versus tumour-bearing mice with orthotopic URCCA4.3 CCA tumours at end of life. (B) Graph compares plasma concentration of PXS-5505 as determined via a LC-MS/MS method developed by Pharmaxis Ltd. and validated over a range of 1.0-500.0 ng/mL in mice bearing orthotopic URCCA4.3 tumors following 1 week of treatment on vehicle or medicated diet formulated with 1120ppm PXS-5505. Datapoints represent quantification of plasma concentration of PXS-5505 per mouse. (C) Graph compares LOX activity in snap frozen aortas as determined by the PXS-5878/Simoa1 from mice bearing established orthotopic URCCA4.3 tumors following 1 week of treatment on vehicle or medicated diet formulated with 1120ppm PXS-5505. Datapoints represent quantification of LOX activity per aorta specimen. Graphs depict mean ±SEM and p-values were determined by Mann-Whitney U test. ** = $p<0.01$ and **** = $p<0.0001$.

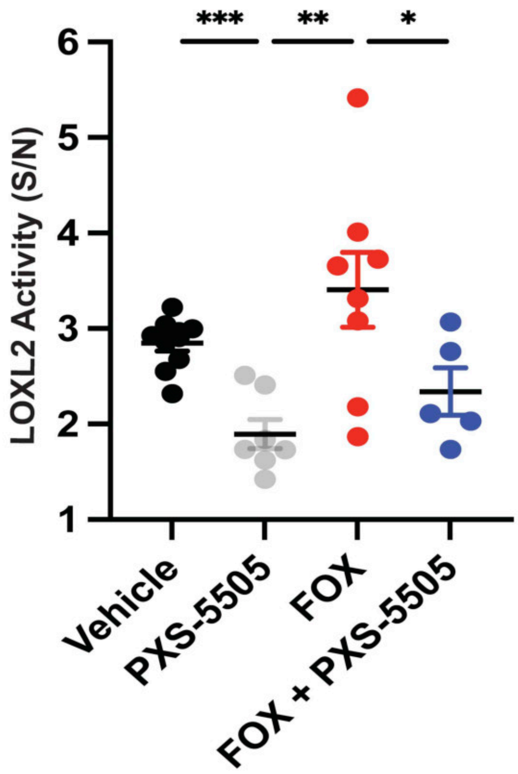

Supplemental Figure 9 Graph compares lysyl oxidase like-2 (LOXL2) isoform activity in snap frozen URCCA4.3 tumours as determined by the PXS-5878/Simoa platform1 after orthotopically implanted mice were treated for 4 weeks as indicated. Datapoints represent quantification of LOXL2 activity per tumour specimen. Graphs depict mean +SEM and p-values were determined by Mann-Whitney U test. * = $p<0.05$, ** = $p<0.01$, and *** = $p<0.001$.

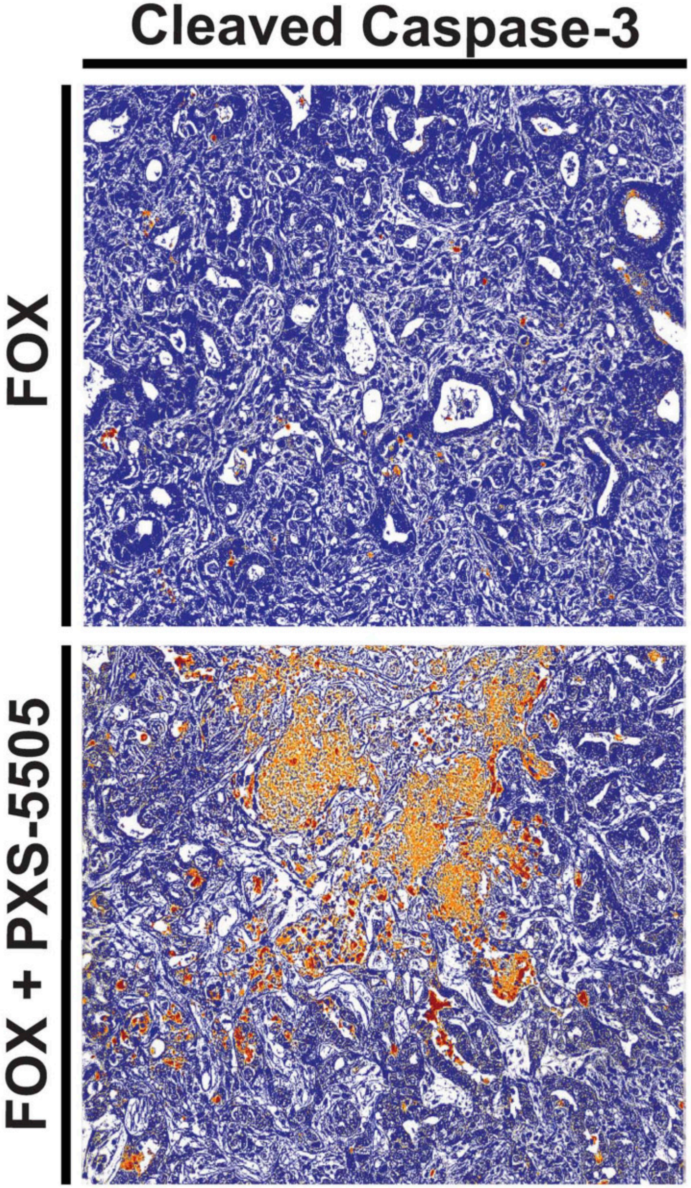

Supplemental Figure 10 Representative images show digital markups with the Aperio Positive Pixel Count algorithm for quantification of CC-3 in tissue sections from orthotopic URCCA4.3 tumors treated for 4 weeks as indicated.

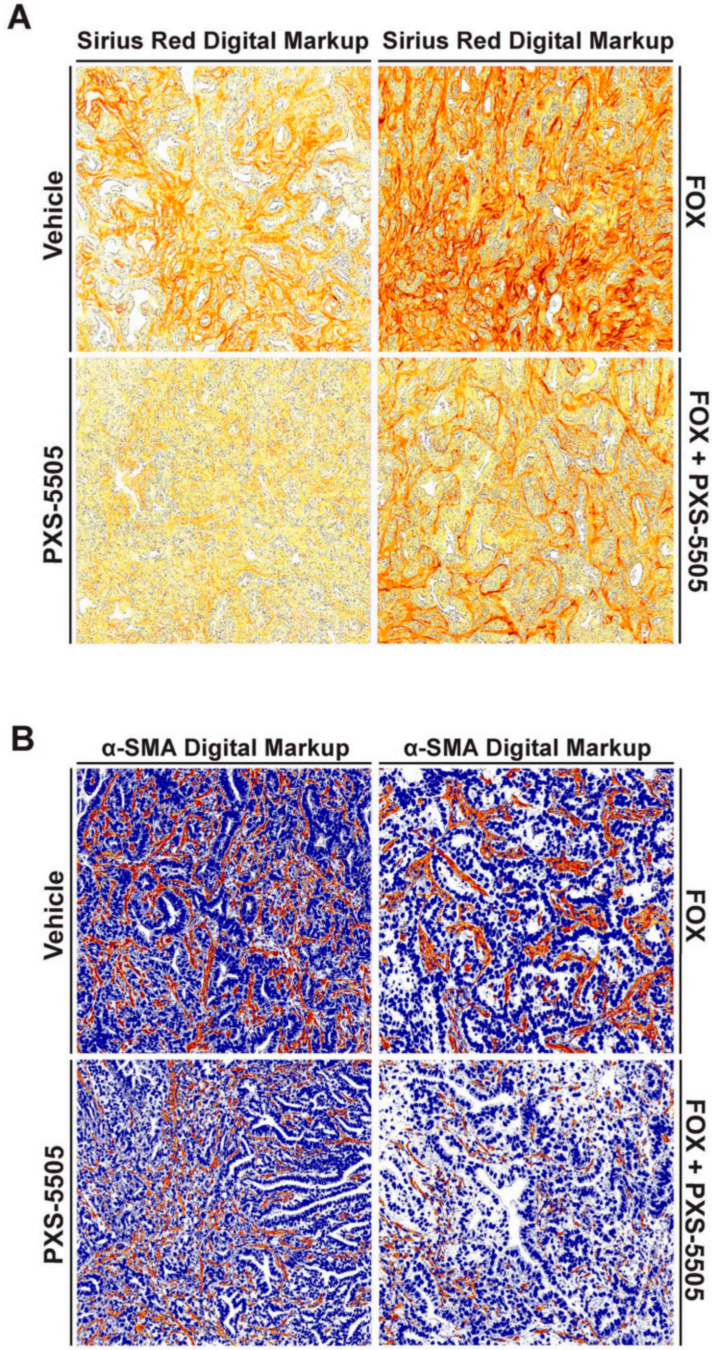

Supplemental Figure 11 (A) Representative images show digital markups with the Aperio Positive Pixel Count algorithm for quantification of sirius red staining in tissue sections after mice with established orthotopic CCA tumors were treated for 4 weeks as indicated. (B) Representative images show digital markups with the Aperio Positive Pixel Count algorithm for quantification of α-SMA IHC staining in tissue sections after mice with established orthotopic CCA tumors were treated for 4 weeks as indicated.

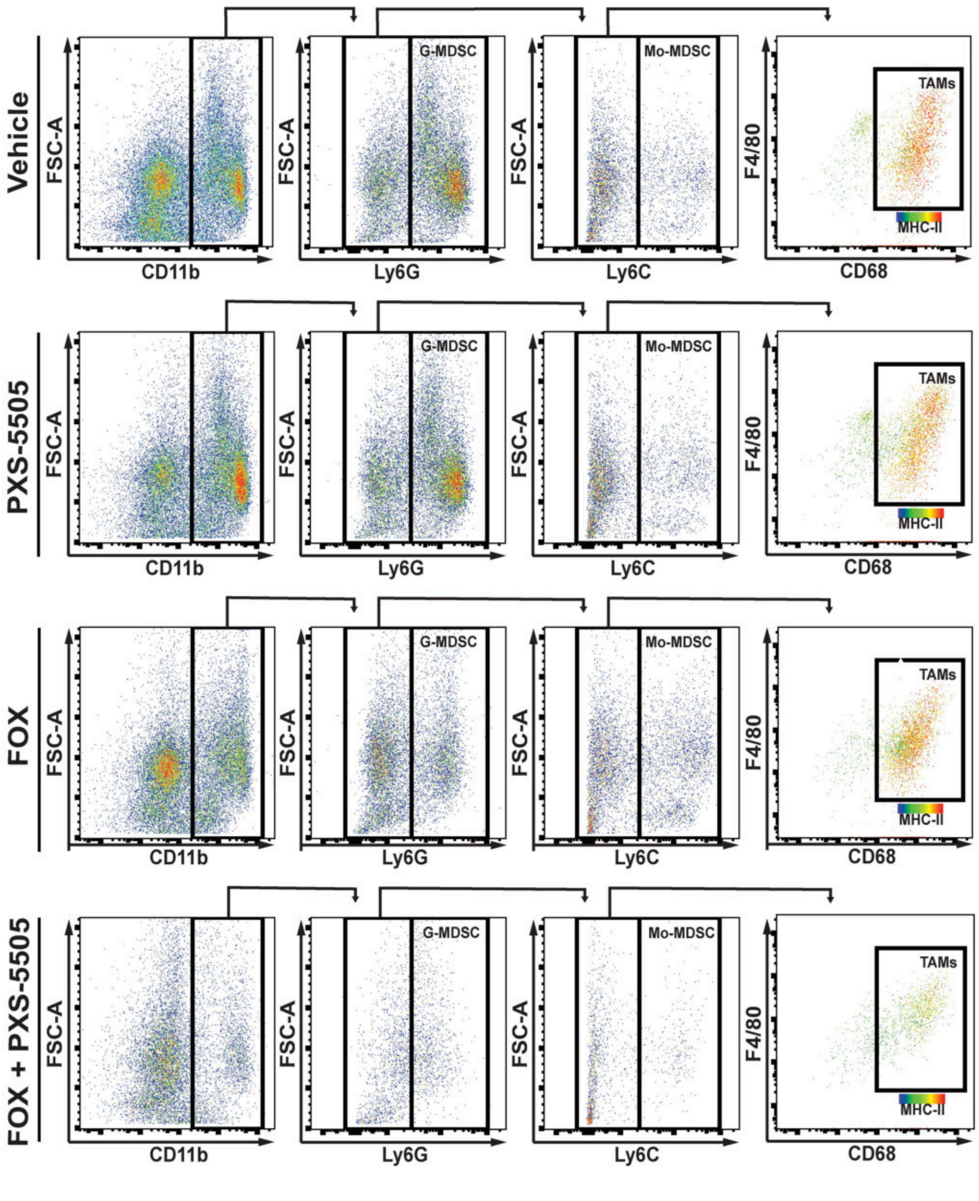

Supplemental Figure 12 Representative flow cytometry plots show gating strategies for identifying myeloid cell subsets in orthotopic URCCA4.3 tumors from groups of mice treated for 3 weeks as indicated.

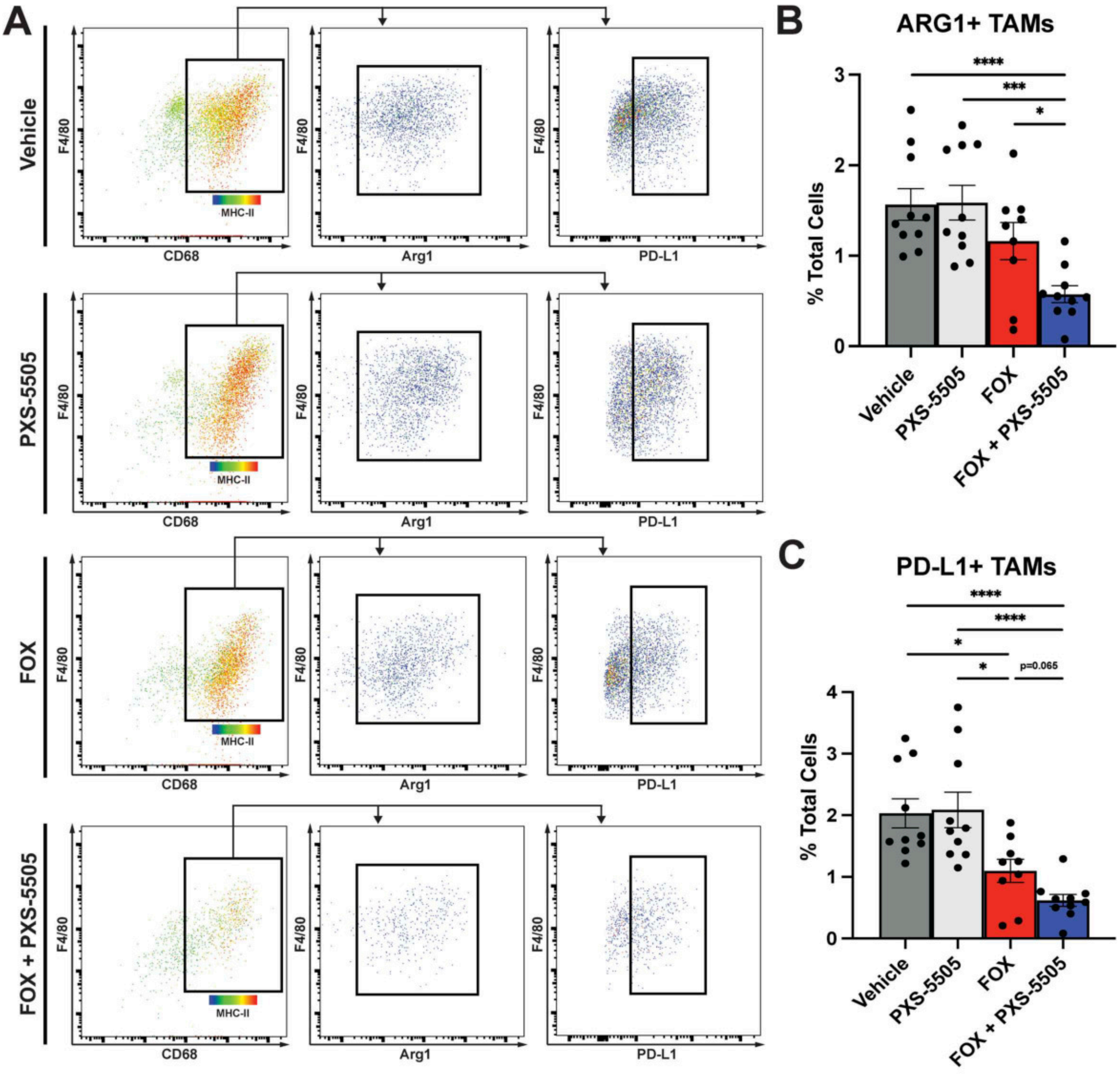

Supplemental Figure 13 (A) Representative flow cytometry plots show gating strategies for identifying TAM subsets expressing arginase-1 (ARG1) and programmed death-ligand 1 (PD-L1) in single-cell suspensions of established orthotopic URCCA4.3 CCA tumors from groups of mice treated for 3 weeks as indicated. (B-C) Graphs compare the frequency of arginase 1 (ARG1) positive (B) and programmed death-ligand 1 (PD-L1) positive (C) TAM by flow cytometry analysis of orthotopic CCA tumors from groups of mice treated as indicated for 3 weeks. n = 9-10 mice per group. Datapoints represent the relative frequency of the indicated cell type(s) per tumor specimen. Graphs depict mean +SEM and p-values were determined by Mann-Whitney U test. * = $p<0.05$, *** = $p<0.001$, and **** = $p<0.0001$.

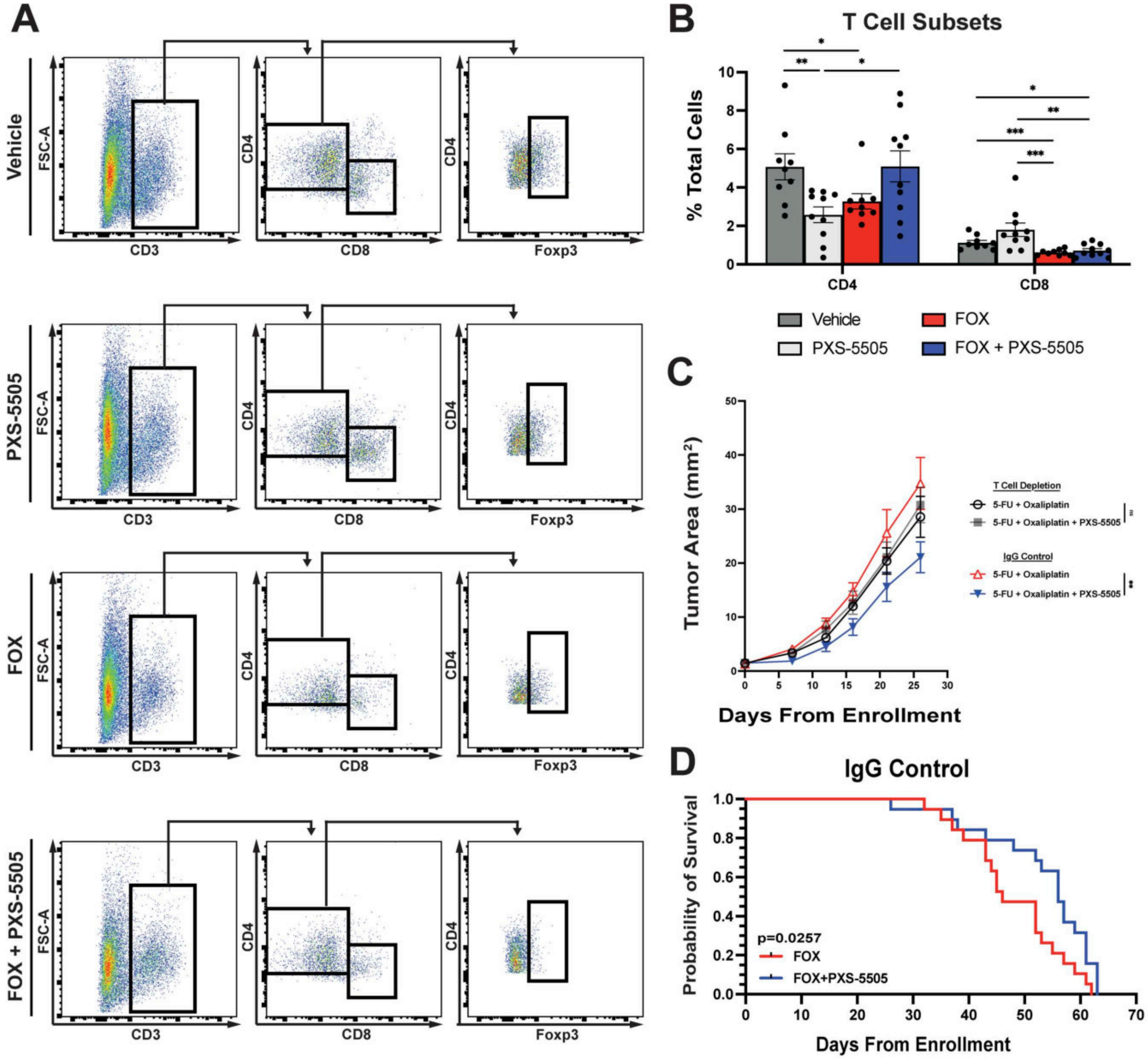

Supplemental Figure 14 (A) Representative flow cytometry plots show gating strategies for identifying T-cell subsets in single-cell suspensions of established orthotopic CCA tumors from groups of mice treated for 3 weeks as indicated. (B) Graph shows the prevalence of tumor-infiltrating T-cell subsets by flow cytometry analysis of orthotopic tumors from mice treated for 3 weeks as indicated. n = 9-10 mice per group. Datapoints represent the relative frequency of the indicated cell type(s) per tumor specimen. Graphs depict mean +SEM and p-values were determined by Mann-Whitney U test. * = p<0.05, ** = p<0.01, and *** = p<0.001. (C) Graph compares tumor growth over time determined by high frequency ultrasonography (US) measurement of the largest cross-sectional tumor area in mice bearing orthotopic CCA tumors treated as indicated. p-value determined by linear regression. n = 9-10 mice per group. ns = not significant. ** = p<0.01. (D) Kaplan-Meier curve compares overall survival of mice with established orthotopic CCA tumors enrolled into treatment groups with FOX (n=19) plus IgG or FOX combined with PXS-5505 plus IgG (n=19). Mice were dosed IP with 500µg of control IgG2b control antibody (BioXcell) prior to tumor implantation followed by 250µg of antibody every 4-5 days. p-value determined by log-rank (Mantel-Cox) test.

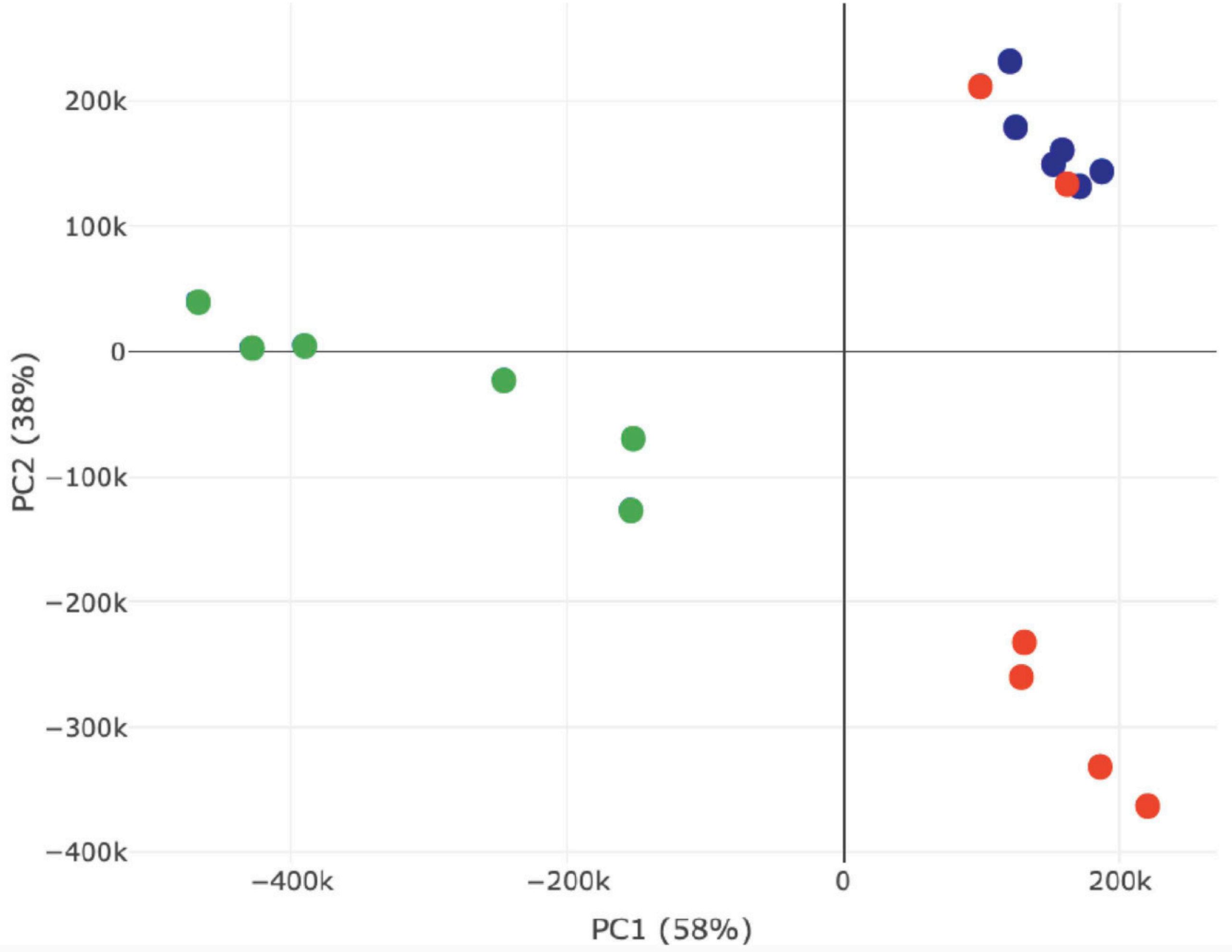

Supplemental Figure 15 Plot shows principle component analysis (PCA) of all differentially expressed protein-coding genes (DEGs) from RNA-seq analysis of TAM (green), TIM (red), and TIM + PXS-5505 (blue) in macrophage invasion assays.

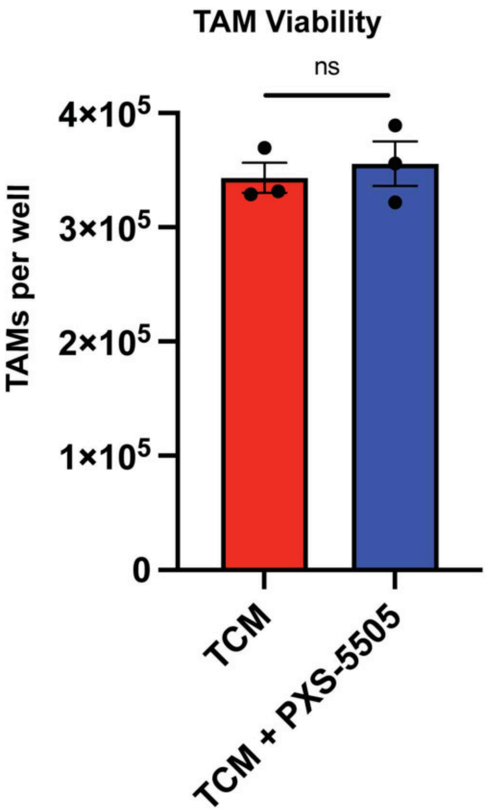

Supplemental Figure 16 Graph compares mean number of cells per well of 6-well plates after culturing 5 x 105 TAMs on thinly layered extracellular matrix (ECM) hydrogel in TCM (n=3) or TCM + PXS-5505 (n=3) for 24 hours. TAMs were quantified by averaging TAMs/μm2 in five random fields at 20X magnification and multiplying by the total well area. Datapoints represent the number of TAMs per well. Graph depicts mean ±SEM and p-values were determined by Mann-Whitney U test. ns = not significant.

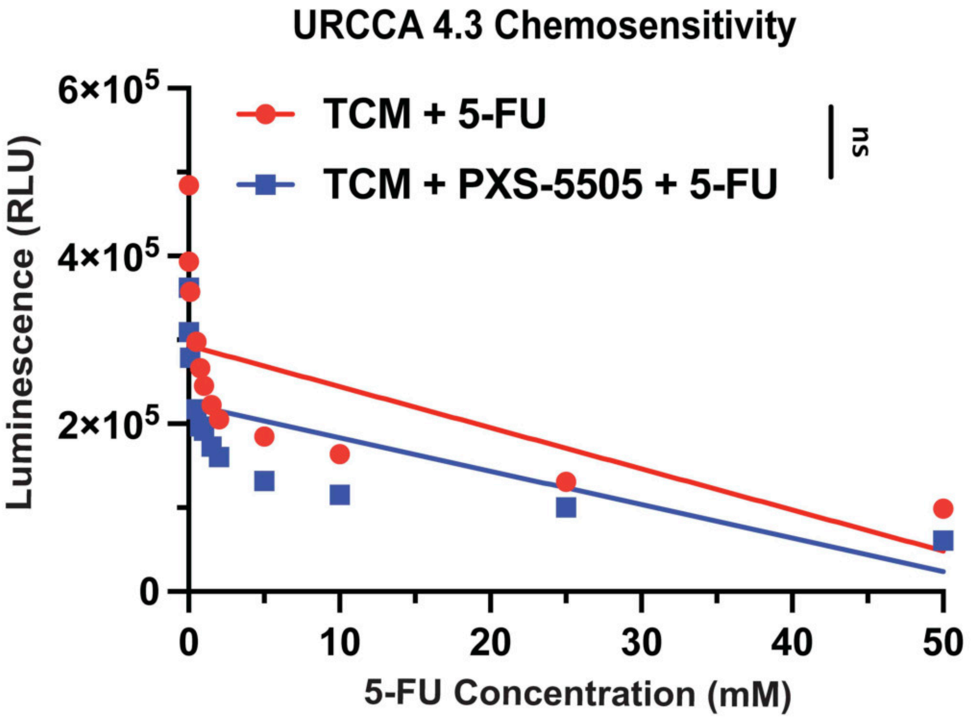

Supplemental Figure 17 Graph compares the viability of 10,000 URCCA 4.3 tumor cells after being seeded in Nunc F96 MicroWell White Polystyrene Plates (ThermoFisher) layered with Cultrex Basement Membrane Extract (R&D Systems) and cultured in CM with or without 1mM PXS-5505 for 24-hours prior to adding fluorouracil and incubating an additional 24-hours. URCCA 4.3 viability was assessed using CellTiter-Glo 2.0 Cell Viability Assays (Promega) and bioluminescence was measured with a Biotek Synergy HTX Multimode Reader. Datapoints depict mean ±SEM and the p-value was determined by simple linear regression. ns = not significant.
