# Supplementary material for: Pan-lysyl oxidase inhibition disrupts fibroinflammatory tumor stroma, rendering cholangiocarcinoma susceptible to chemotherapy
Source: Hepatol Commun. 2024 Aug 5;8(8):e0502. doi: 10.1097/HC9.0000000000000502 (PMC11299993; doi:10.1097/HC9.0000000000000502)
Supplement: Supplementary file 4 [file hc9-8-e0502-s004.docx]

**Supplemental Table 8**

| **Characteristics** | **FOX**  **(n = 20)** | **FOX + PXS-5505 (n = 21)** | **Statistical Analysis** |
| --- | --- | --- | --- |
| **Female Sex (n, %)** | 11 (55%) | 11 (52%) | p = 0.87 |
| **Primary Tumor Size (mm^2^) (mean, SD)** | 2.896 (2.94) | 2.142 (1.23) | p = 0.95 |
| **Primary Tumor Location**  Left Lobe  Right Lobe  Middle Lobe  Caudate Lobe | 6  8  6  0 | 11  6  4  0 | p = 0.34 |
